# Applications of EEG indices for the quantification of human cognitive performance: A systematic review and bibliometric analysis

**DOI:** 10.1371/journal.pone.0242857

**Published:** 2020-12-04

**Authors:** Lina Elsherif Ismail, Waldemar Karwowski

**Affiliations:** Department of Industrial Engineering and Management Systems, Computational Neuroergonomics Laboratory, University of Central Florida, Orlando, FL, United States of America; National University of Sciences and Technology, PAKISTAN

## Abstract

**Background:**

Neuroergonomics combines neuroscience with ergonomics to study human performance using recorded brain signals. Such neural signatures of performance can be measured using a variety of neuroimaging techniques, including functional magnetic resonance imaging (fMRI), functional near-infrared spectroscopy (fNIRS), and electroencephalography (EEG). EEG has an excellent temporal resolution, and EEG indices are highly sensitive to human brain activity fluctuations.

**Objective:**

The focus of this systematic review was to explore the applications of EEG indices for quantifying human performance in a variety of cognitive tasks at the macro and micro scales. To identify trends and the state of the field, we examined global patterns among selected articles, such as journal contributions, highly cited papers, affiliations, and high-frequency keywords. Moreover, we discussed the most frequently used EEG indices and synthesized current knowledge regarding the EEG signatures of associated human performance measurements.

**Methods:**

In this systematic review, we analyzed articles published in English (from peer-reviewed journals, proceedings, and conference papers), Ph.D. dissertations, textbooks, and reference books. All articles reviewed herein included exclusively EEG-based experimental studies in healthy participants. We searched Web-of-Science and Scopus databases using specific sets of keywords.

**Results:**

Out of 143 papers, a considerable number of cognitive studies focused on quantifying human performance with respect to mental fatigue, mental workload, mental effort, visual fatigue, emotion, and stress. An increasing trend for publication in this area was observed, with the highest number of publications in 2017. Most studies applied linear methods (e.g., EEG power spectral density and the amplitude of event-related potentials) to evaluate human cognitive performance. A few papers utilized nonlinear methods, such as fractal dimension, largest Lyapunov exponent, and signal entropy. More than 50% of the studies focused on evaluating an individual’s mental states while operating a vehicle. Several different methods of artifact removal have also been noted. Based on the reviewed articles, research gaps, trends, and potential directions for future research were explored.

**Conclusion:**

This systematic review synthesized current knowledge regarding the application of EEG indices for quantifying human performance in a wide variety of cognitive tasks. This knowledge is useful for understanding the global patterns of applications of EEG indices for the analysis and design of cognitive tasks.

## 1. Introduction

The discipline of human factors and ergonomics investigates the interactions between humans, machines, the environment, and technology while considering human capabilities and limitations to ensure safe and satisfying working environments [1–4]. Traditional techniques and methods evaluated work tasks in a subjective manner, using a variety of qualitative approaches [5–7]. Such approaches do not allow for adequate analysis of the complex interactions between the cognitive, perceptual, and physical aspects of working with modern technology [3, 8–11] nor do they allow us to model and quantify the complex relationship between the human mind and technology [11].

Recent advances in artificial intelligence, autonomous systems, and modern industrial automation such as digital manufacturing (i.e., Industry 4.0) have created the need for today’s human operators to collaborate with sophisticated and dynamically changing technological environments that require high levels of cognitive and perceptual [12, 13]. Therefore, a deeper understanding of human performance by considering the human brain at work is needed. The pioneering concept known as neuroergonomics was first introduced by Parasuraman et al. [14, 15]. This study of the brain and behavior at work applies methods and tools from neuroscience to study brain signatures of human performance in everyday life activity [16]. Neuroergonomics research aims to expand our understanding of the neural mechanisms underlying cognitive and motor functioning with a focus on real-world applications. Cognitive ergonomics focuses on mental processes such as perception, information processing, and decision-making that could be applied with immobile participants [3, 11, 16, 17].

The human brain, a single organ that coordinates all bodily functions and controls every aspect of the body, is composed of over 100 billion neurons [18]. Communication between neurons occurs via electrical signals whose flow results in the generation of an electrical current, which subsequently creates wave patterns termed “brain signals.” Different classifications of brain signals are available in the literature [19, 20], but the most widely used taxonomy is based on the frequencies of the brain waves measured in hertz (Hz), as follows: delta (δ: 0.5–4 Hz), theta (θ: 4–8 Hz), alpha (α: 8–13 Hz), beta (β: 13–30 Hz), and gamma (γ: 30–150 Hz) [21]. Different brain functions are associated with different lobes of the brain. For example, the frontal lobe is associated with planning, voluntary movement, emotion, reasoning, and problem-solving; the parietal lobe is associated with memory, hearing, vision, sensory, and motor function; and the temporal lobe is associated with the recognition and perception of auditory stimuli and language. Finally, the occipital lobe is responsible for processing visual information.

The number of experimental studies focusing on neuroergonomics has increased substantially with the emergence of neuroimaging techniques [22], which are based on measuring neural activity rather than changes in cerebral blood flow or voltage fluctuations resulting from ionic current [23–25]. EEG has both advantages and disadvantages compared with other neuroimaging measures, which render it both useful and challenging in neuroergonomics applications. The main advantages include (1) a high degree of temporal resolution [26], (2) portability for use in real-life environments, and (3) affordability [27]. However, EEG techniques also exhibit three significant drawbacks: (1) low spatial resolution [21], (2) the existence of undesired nonbrain signals or “artifacts” [19, 28], and (3) the long preparation time required for setup [25]. Despite these challenges, recent advances in EEG technology have led to the development of wireless EEG systems that allow participants to conduct ongoing work without interference [29, 30] and that apply dry electrodes instead of using wet systems, thereby decreasing preparation time [31–33]. Furthermore, automatic artifact detection software [34] has been developed to improve signal quality. EEG analysis methods are categorized into the time domain, frequency domain, time-frequency domain, and nonlinear methods. EEG indices are reliable indicators that reflect spontaneous activity in the brain. In this regard, we found it is essential to explore the research into EEG indices in cognitive work. The main aim of the current article is to systematically review the application of EEG indices in the context of neuroergonomics to understand the current state of knowledge based on related articles. Based on predefined research questions, we aimed to comprehensively review the articles at the macro and micro scales by identifying and summarizing various article characteristics, including journals, research topics, highly cited papers, and highly frequent keywords. Using bibliometric methods, we mapped the interrelationships among journal publications, citations, and keywords. Based on the bibliometric analysis, we then discuss the global trends, research gaps, and potential future directions. The present study follows up on a prospective review by Rabbi et al. [35], which provided a useful foundation for other scholars in understanding cognitive work based on EEG indices in 15 publications from 1994 to 2008. Our current study systematically explores the use of EEG indices in quantifying human performance in 143 cognitive studies published between 2000 and 2019. Our literature analysis is the first to be conducted at the macro and micro scales. The macro-scale analysis was used to identify general trends in the application of EEG indices in cognitive work, whereas the micro-scale approach allowed us to analyze and compare the findings of individual studies.

In the next section, we provide the research methodology and criteria for inclusion and exclusion of studies. **Section 3** provides the macro-scale analysis of the literature. **Section 4** provides the micro-scale analysis of the literature, where we discuss detailed applications of EEG indices in cognitive work in the context of neuroergonomics. **Section 5** provides a statistical summary and major findings. **Section 6** provides the research gaps and future directions. In the final section, we reach the conclusions.

## 2. Methods

### 2.1 Review standards

This systematic review was conducted using the guidelines for preferred reporting items for systematic reviews and meta-analyses (PRISMA) [36–38]. PRISMA is a structured guideline that helps ensure reliable and meaningful review results. The guideline consists of 27 checklist items that help researchers prepare and report evidence accurately and reliably to improve the quality of research [37]. The selection of the articles reviewed herein was based on both specific research questions and a search strategy aimed at reducing the effect of research expectations on the current review.

### 2.2 Research questions

The following research questions (RQ) were defined according to the objectives of the study:

- RQ1: What is the dominant EEG index used to quantify human performance in cognitive work?
- RQ2: What are the different applications of EEG, methods of feature extraction, and methods of artifact removal that have been addressed to date pertaining to cognitive work?
- RQ3: What are the current limitations to characterizing and predicting human performance using EEG data?

### 2.3 Search strategy

Comprehensive literature searches were independently conducted using Web-of-Science and Scopus databases. First, we applied the following search terms and Boolean operators: “electroencephalography” OR “EEG” AND “cognitive work” OR “cognitive task” OR “cognitive functions” OR “mental states.” Searches were restricted to articles published from 2000 to 2019. The final search performed for the present study was conducted on October 21, 2019. Articles related to neuroscience, brain diseases, clinical studies, and participants in pathological conditions were outside the scope of the current review. This search resulted in a total of 1767 articles; then, to ensure we collected all relevant articles during the literature search, the reference lists of the candidate articles (n = 1767) were also reviewed, which resulted in 44 additional articles. Subsequently, duplicate articles were removed, resulting in 1405 records.

### 2.4 Study selection

To maintain the focus on neuroergonomics studies, more keywords were applied, as follows:

“electroencephalography” OR “EEG” AND “cognitive work” OR “cognitive task” OR “cognitive functions” OR “mental states” AND “neuroergonomics” OR “human factors” OR “human performance” OR “ergonomics” OR “safety” OR “hazard” OR “error” OR “accidents” OR “fatigue” OR “workload” OR “effort” OR “vigilance” OR “attention” OR “alertness” OR “drowsiness” OR “emotion” OR “stress” OR “decision making”. The use of these keywords narrowed the resulting studies to meet our focus and led to the exclusion of 895 articles. Subsequently, the titles and abstracts of the articles were screened. After independently reviewing all titles and abstracts of the remaining articles, two researchers (LEI and WK) independently reviewed the full text of 233 articles for inclusion and exclusion criteria. Any disagreements were resolved by consensus.

### 2.5 Criteria for inclusion and exclusion

**Fig 1:**
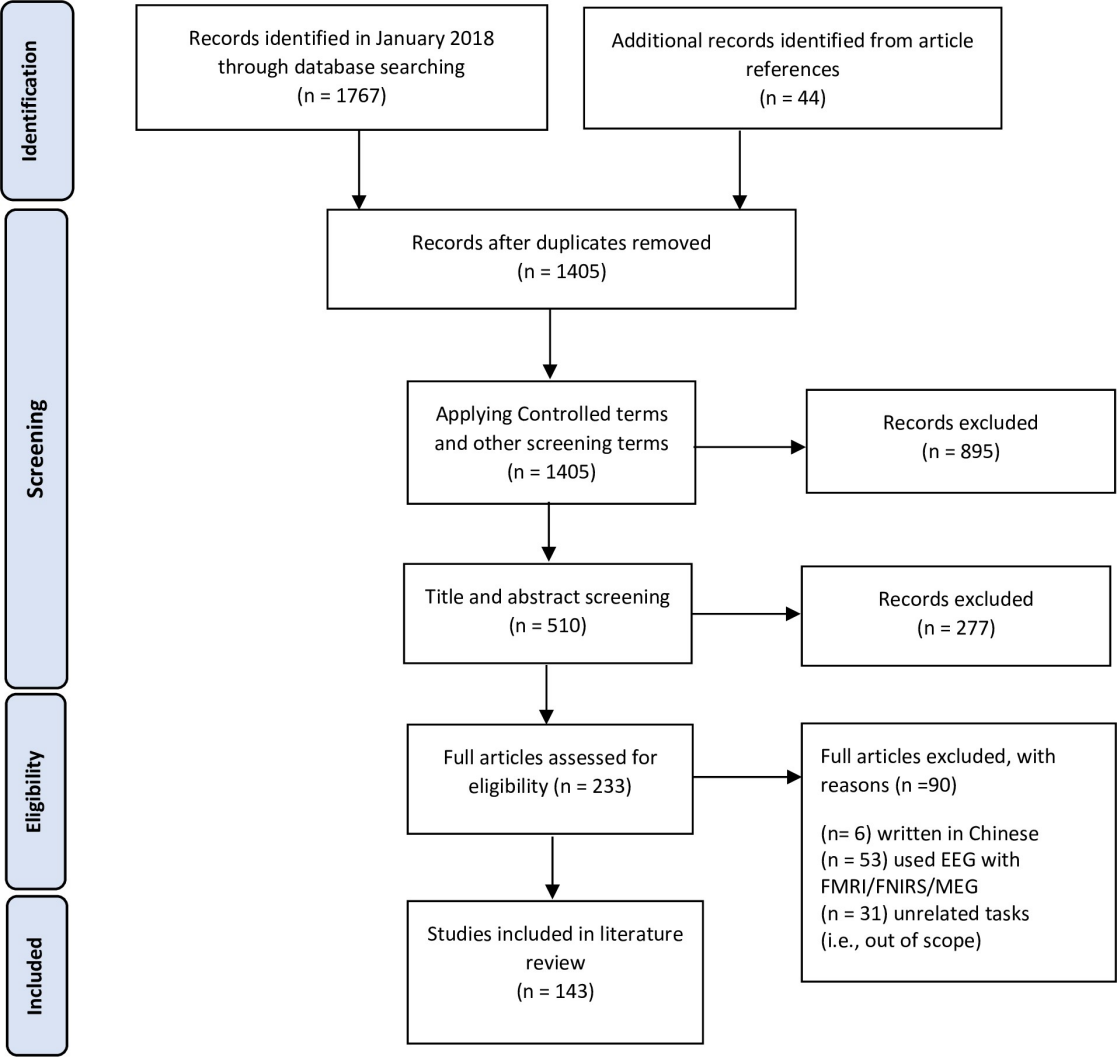
Selection process for the cognitive work studies included in the present review according to PRISMA guidelines.

To meet the eligibility criteria, only published articles with the following criteria were included: (a) English language articles, (b) experiments involving human subjects, (c) experimental studies on healthy participants, and (d) content from peer-reviewed journals, conference publications, textbooks, and reference books. Articles with the following features were excluded: (a) studies that were not associated with cognitive tasks, (b) studies that focused on brain diseases or neural disorders, and (c) studies that used EEG combined with other neuroimaging techniques, such as functional magnetic resonance imaging (fMRI), functional near-infrared spectroscopy (fNIRS), or magnetoencephalography (MEG). Accordingly, 6 studies were eliminated because the full text was available only in Chinese, 53 studies were discarded because they combined EEG with other neuroimaging techniques (as the current study focused solely on EEG indices), and 31 studies were outside the scope of the current study. The selection process and the findings of the literature search are summarized in the PRISMA flowchart **Fig 1.**

### 2.6 Data collection and summary measures

Relevant information was extracted for each study, including all physiological measurements used; EEG indices; experiments in cognitive tasks; and methods of artifact removal, feature extraction, selection, and classification methods (see S1 Appendix).

### 2.7 Data extraction and synthesis

A total of 143 articles were eligible for this systematic literature review. Tasks selected in the current study involved cognitive function with minimal physical requirements.

## 3. Macro-scale analysis of the literature

**Fig 2:**
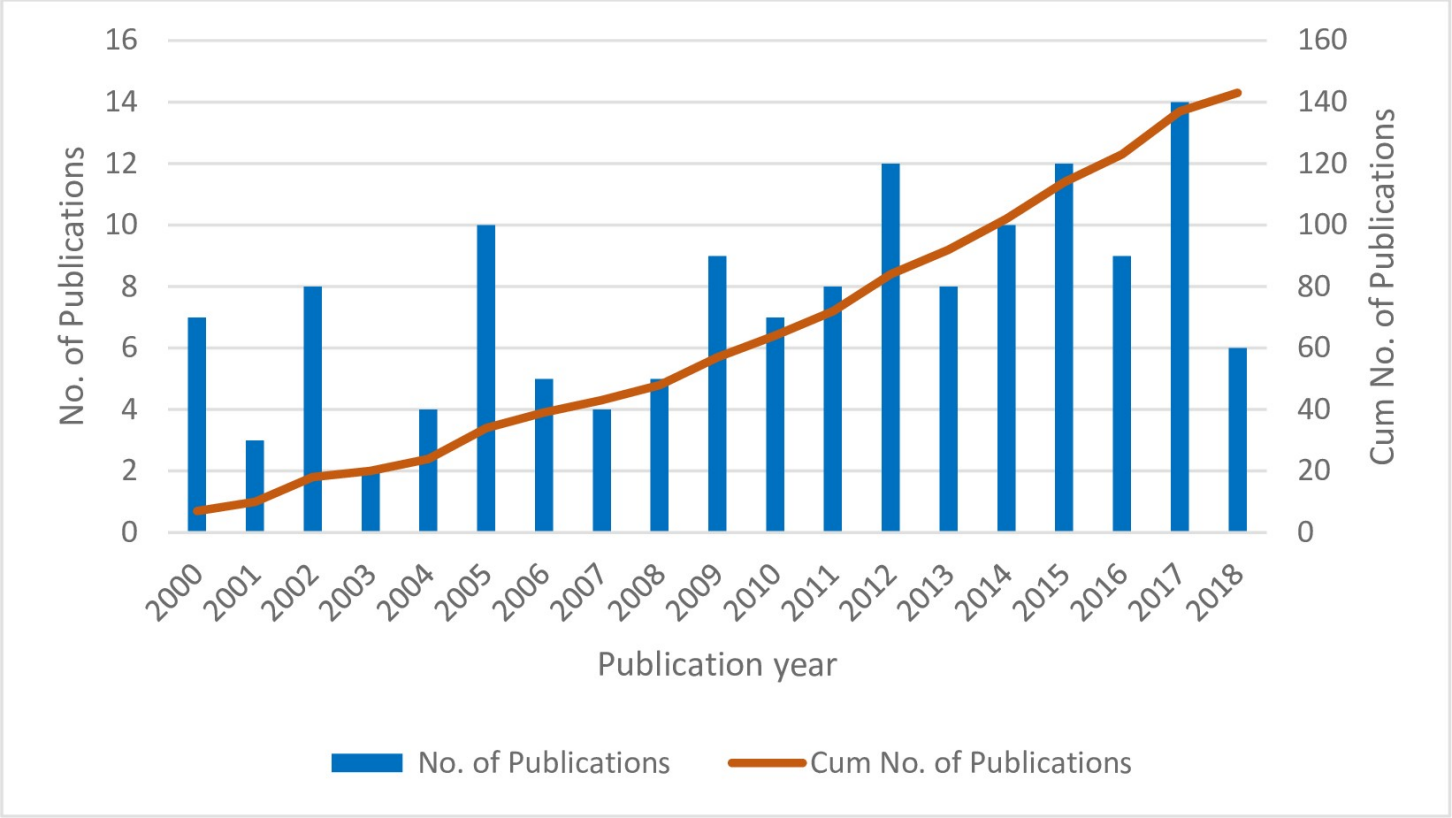
Temporal distribution of studies of EEG indices in cognitive work.

Bibliometrics is a research method that considers the bibliometric characteristics of a research article and provides a useful tool to quantitatively analyze a specific research area [39]. Statistical methods are applied to explore various characteristics of the research article. Bibliometric analysis can provide (1) different aspects of a unique research topic, (2) a macro-scale analysis of literature, (3) a micro-scale analysis of literature, (4) research trends, and (5) research gaps for future directions. The bibliometric data were first analyzed at the macro scale to determine the trends and classify performance measures. **Fig 2** demonstrates the temporal distribution of studies of EEG indices in cognitive work published from 2000 to 2018. The data shows an increasing trend in the applications of EEG indices in cognitive work, with the highest number of publications in 2017.

### 3.1 Analysis of main journals and conference publications

To visualize and analyze the bibliometric data, we used VOSviewer software [40] for scientometric analysis. VOSviewer software allows us to map the bibliometric data as a network and create various visualization maps, such as keyword co-occurrence, co-authorship, citation, co-citation, and bibliographic coupling maps. As defined by Van and Waltman [40], “each circle in the map represents a term, and the size of the circle and font represents the activity of the term.”

**Fig 3:**
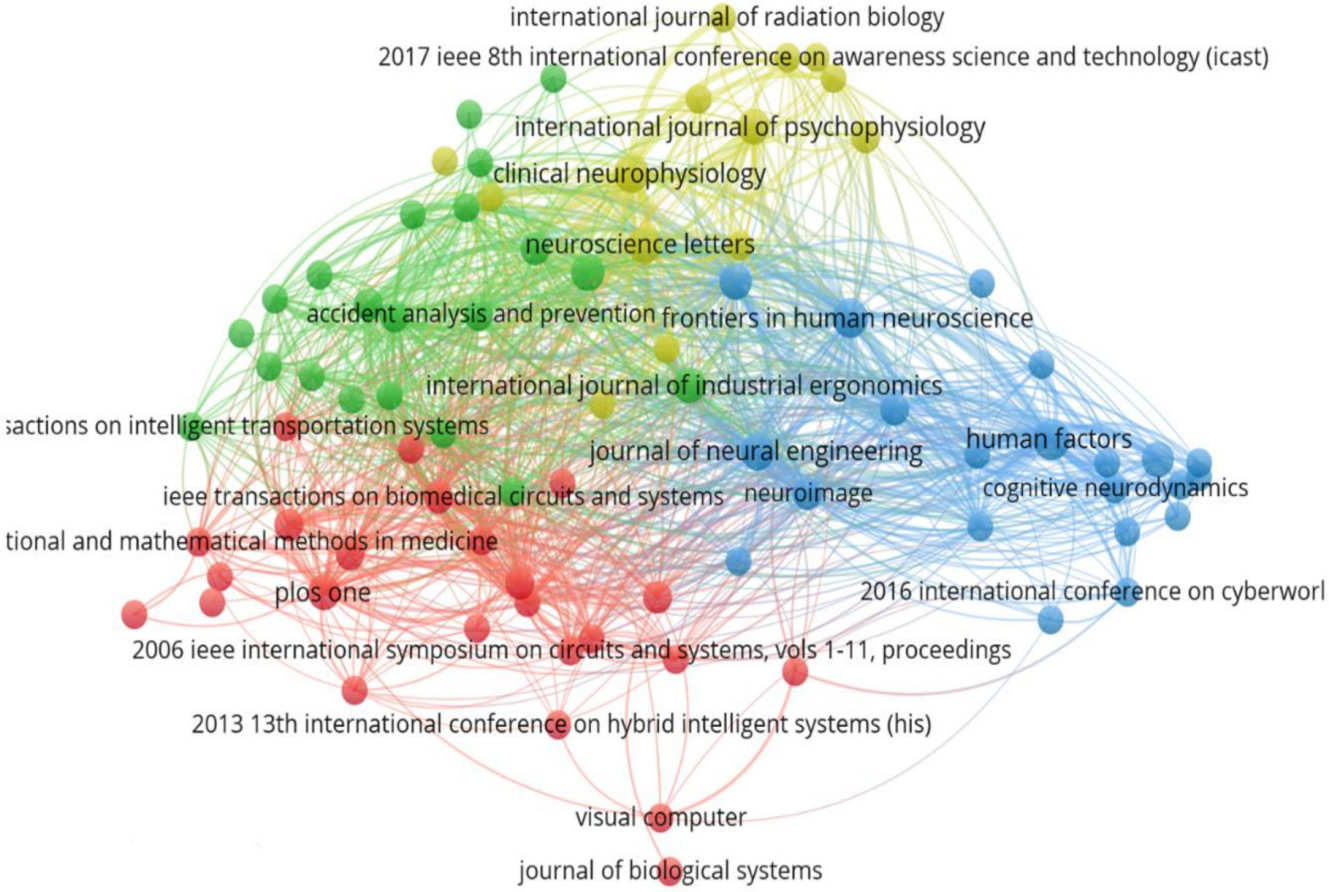
Bibliographic coupling based on the source.

**Fig 4:**
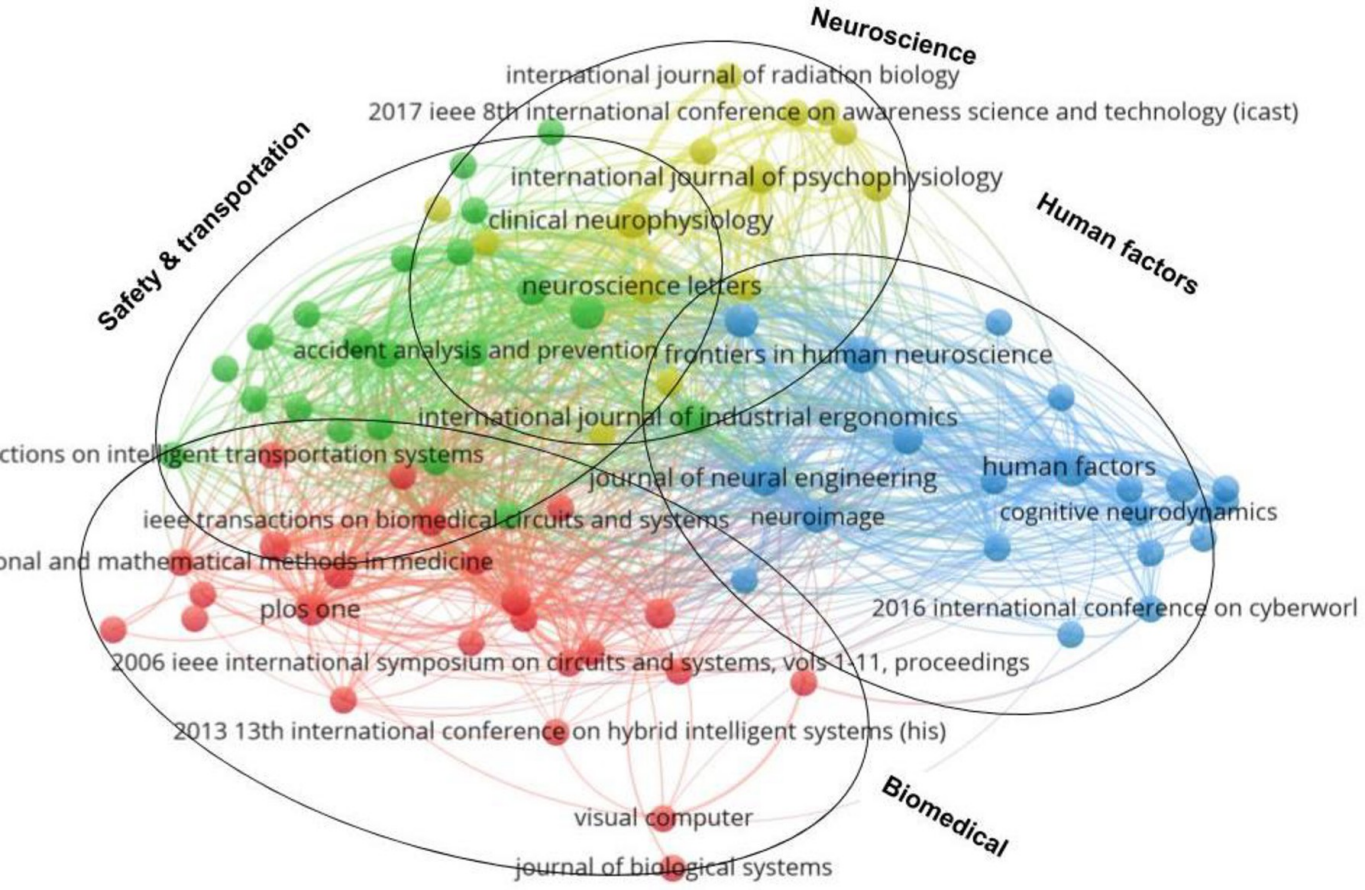
Classification of bibliographic coupling based on the source.

**Fig 3** shows a map of bibliographic coupling based on the cited source, with 87 sources. Each circle represents a source (journal or conference paper), and the size and the font are indicators of the source activity. The larger the circle, the more active the source is in the field and vice versa. The distance between two circles represents the degree of association between two sources. The shorter the distance, the stronger the correlation between the sources (and vice versa). The thickness of the links between the sources indicates the strength of the co-citation relationship. Strong co-citation between sources generates clusters. As indicated in **Fig 4**, EEG indices in cognitive work have been applied in various areas, including human factors, ergonomics, biomedical engineering, neural engineering, human neuroscience, clinical neuroscience, psychophysiology, information science, aviation, and intelligent systems.

In terms of the number of publications, studies published by *Human Factors* (n = 7), *Frontiers in Human Neuroscience* (n = 5), *Clinical Neurophysiology* (n = 5), and *PLoS One* (n = 4) have contributed most to cognitive work assessment based on EEG indices. Journal publishing information can be classified into four clusters, as shown in **Fig 4.**

### 3.2 Analysis of journal co-citations

**Fig 5:**
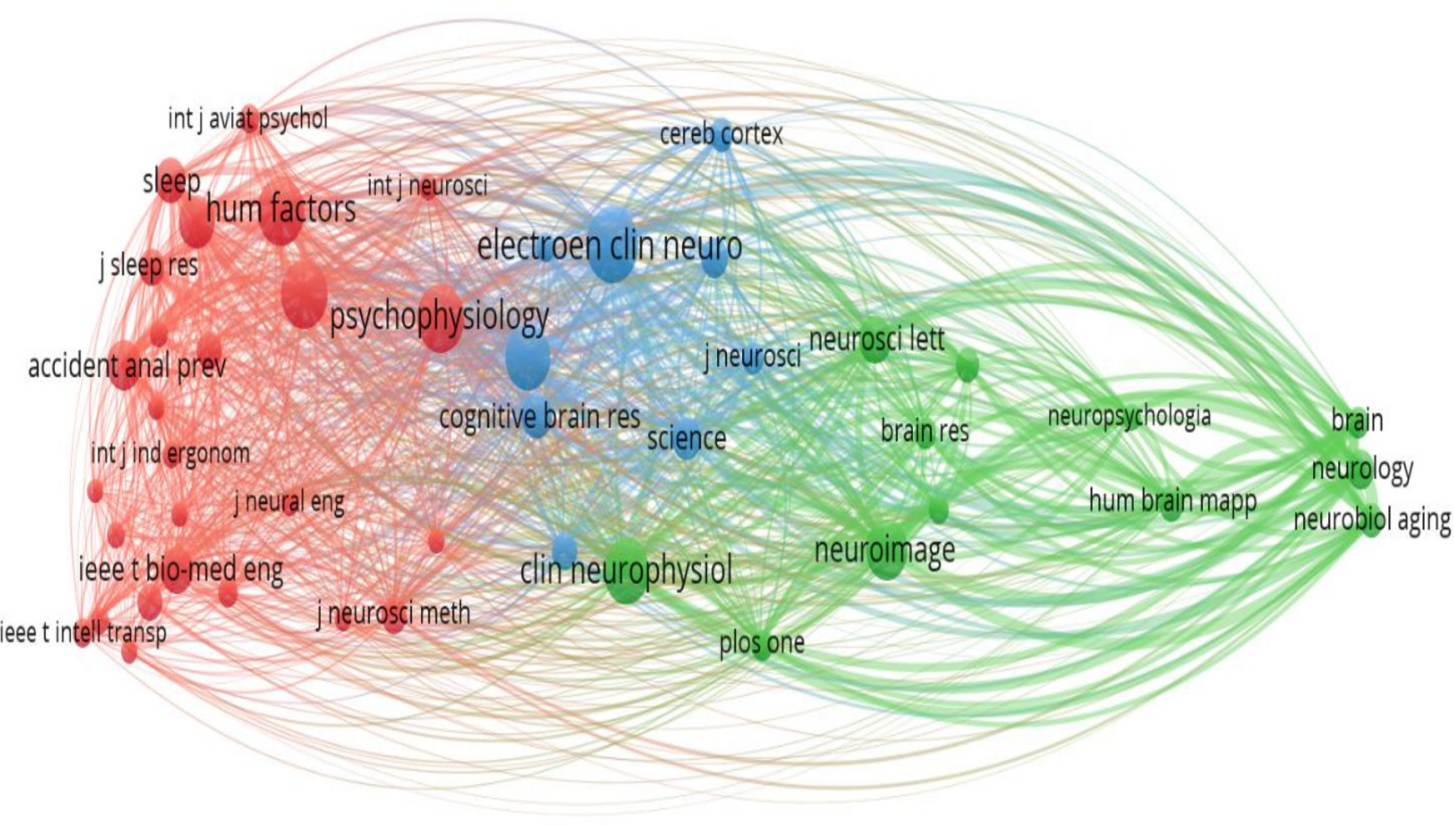
Map of co-citation relationships between publications.

**Fig 5** shows the co-citation map, which represents the relationships between publications aggregated over the source of origin. A minimum of 20 citations was set as a threshold to maintain transparency of the map. A total of 47 journal sources met the threshold. The red cluster is mainly represented by *Human Factors*, *Accident Analysis and Prevention*, *Psychophysiology*, *Sleep*, *Biomedical Engineering*, *Ergonomics*, *Neural Engineering*, and *Sleep Research*. The blue cluster is mainly represented by *Electroencephalography and Clinical Neurophysiology*, *Cognitive Brain Research*, *Cerebral Cortex*, *Science*, and *NeuroReport*.

The green cluster is mainly represented by *Human Brain Mapping*, *NeuroImage*, *PLoS One*, *Neurobiology*, *Brain Research*, *Neuroscience Letters*, and *Neurology*.

### 3.3 Analysis of main affiliations

**Fig 6:**
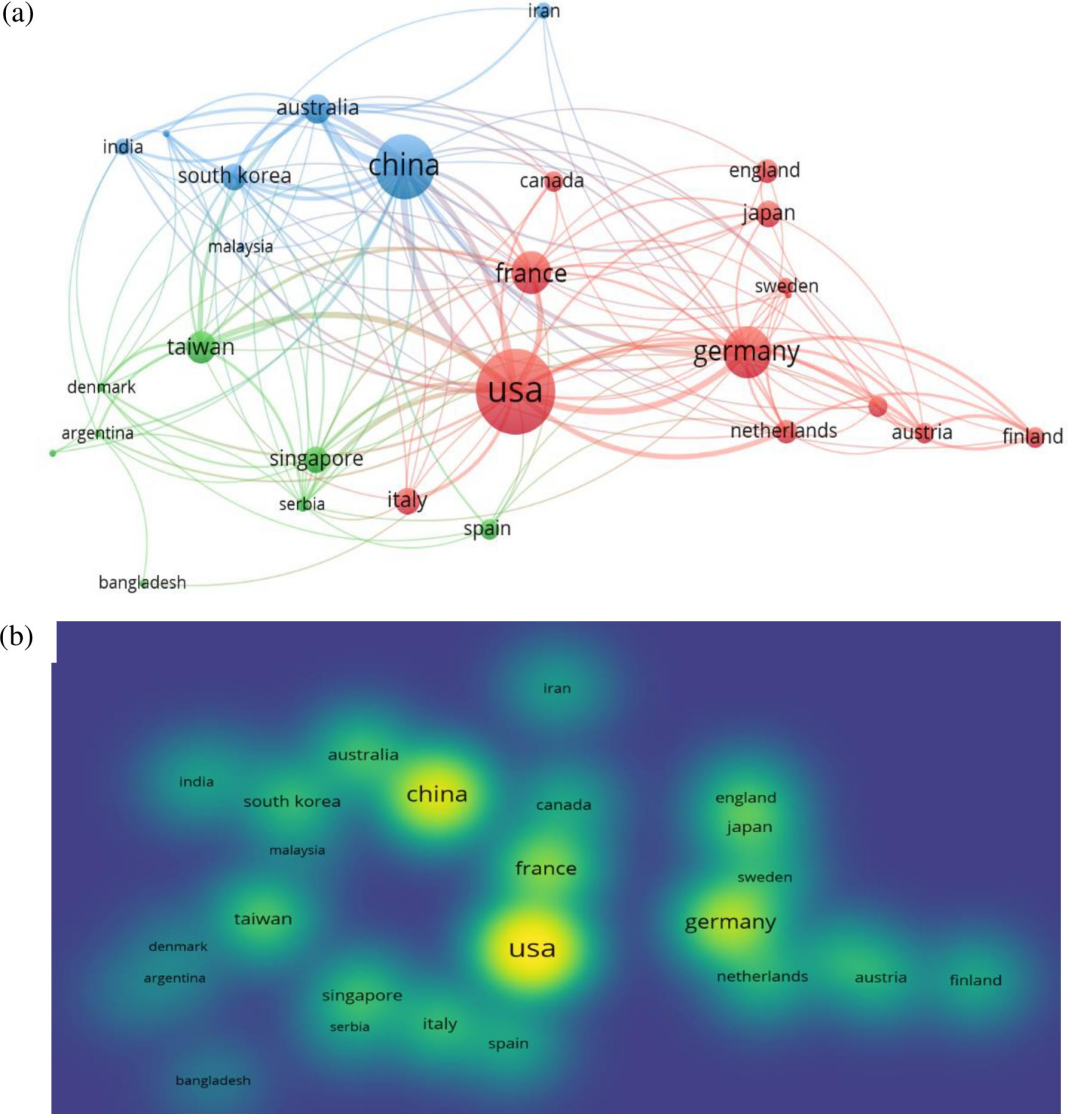
Main affiliations in cognitive research (a) network visualization and (b) density visualization.

**Fig 6** maps co-citations by country. Overall, the map demonstrates the predominance of publications on applications of EEG indices in cognitive work by researchers in the United States, China, Germany, and France.

### 3.4 Analysis of high-frequency keywords

**Fig 7:**
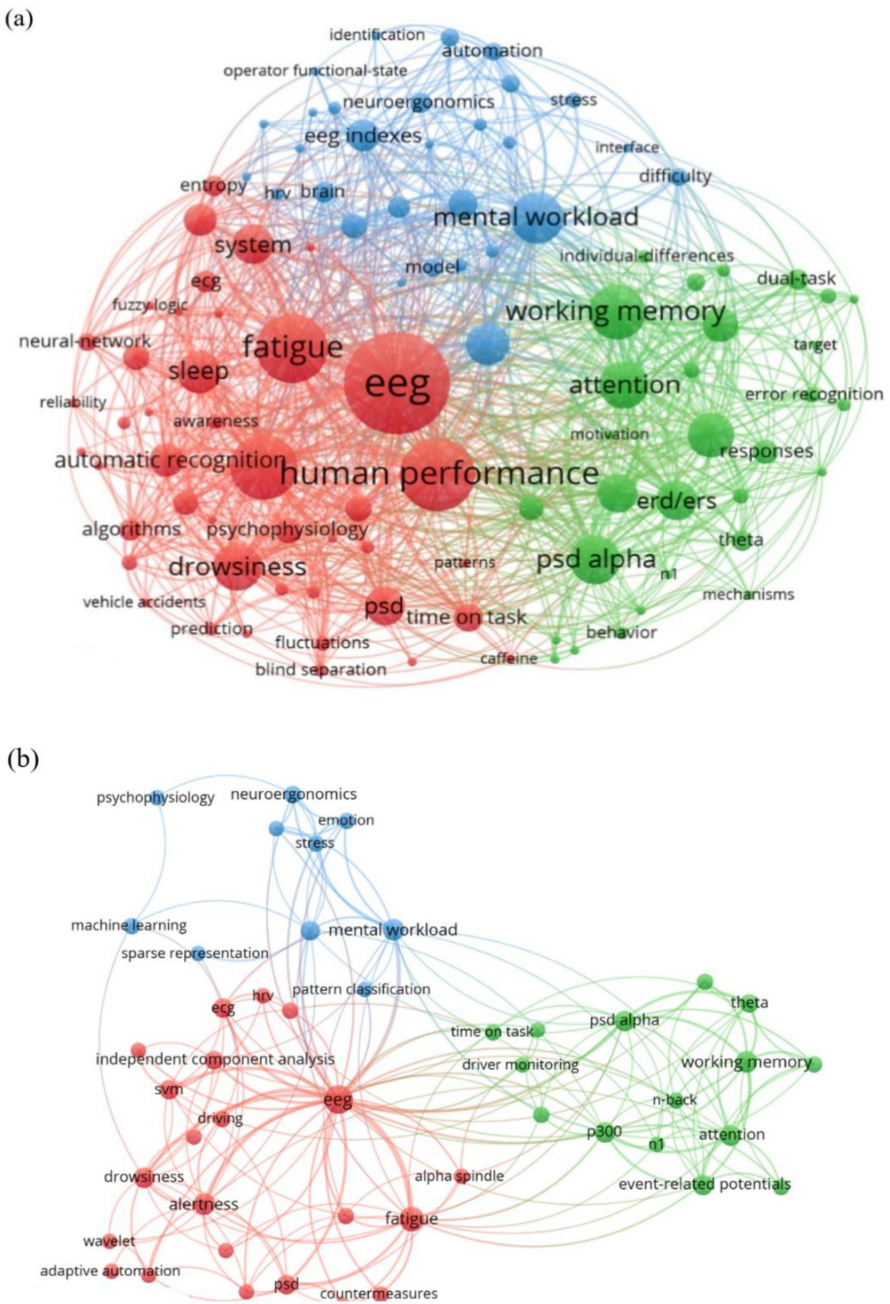
Network visualization map of co-occurrence (a) title and abstract keywords, and (b) author keywords.

To better illustrate the general macro-scale applications of EEG indices in cognitive work, keywords in article titles and abstracts were analyzed by developing a co-occurrence map. The map shows high-frequency keywords and the relationships between keywords. Circles represent a specific key term, and the size of the circle indicates the frequency of that term. The distance between the two circles indicates the co-occurrence of their corresponding key terms in publications. The threshold of keyword frequency was set at 2. Out of 596 keywords, 103 met the threshold, as described in **Fig 7A** We also developed a map for author keywords only, where 56 keywords met the threshold, as shown in **Fig 7B**.

**Fig 8:**
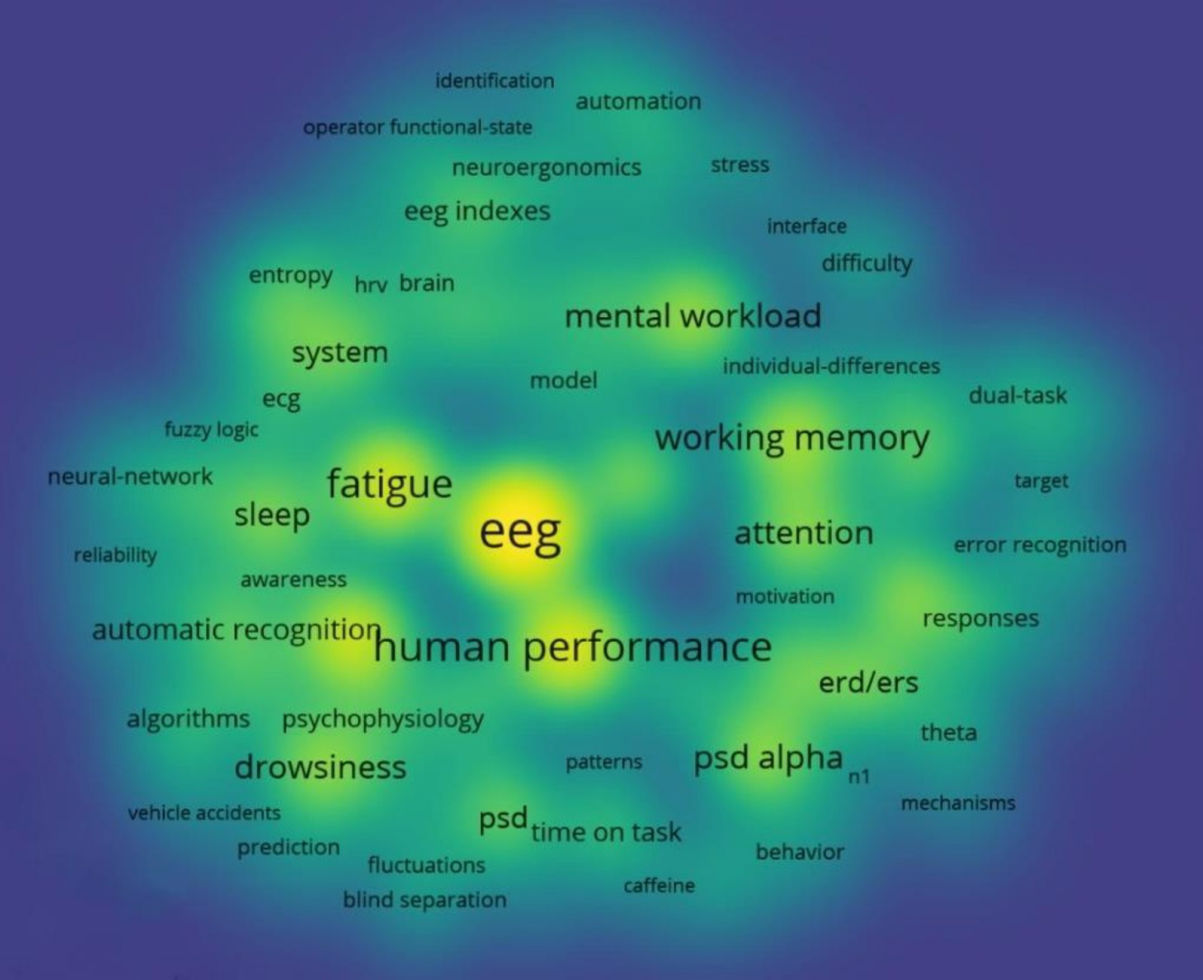
Density view of a network visualization map showing co-occurrence of title and abstract keywords.

We created a density view of the high-frequency keywords, as shown in **Fig 8**, in which a lighter color (i.e., closer to yellow) indicates higher occurrence of a key term. The core keywords of the reviewed articles included “*EEG*,” and “*human performance*.” Performance measures that attracted broad attention included “*fatigue*,” with its related key terms of “*alertness*,” “*drowsiness*,” “*sleep*,” and “*attention*”; “*mental workload*” with its related key term “*difficulty*”; and “working memory.” The map shows that power spectral density (“*PSD*”), and its related key terms such as “*alpha P*SD,” “*alpha spindle*,” “*theta*,” “*ERD/ERS*,” and “*synchronization*,” has frequently been applied as an EEG index for analyzing EEG data based on the frequency domain. Furthermore, “*event-related potentials*,” and its related key terms such as “*P300*” and “*n1*,” has been applied to analyze EEG data based on the time domain. The task of vehicle driving with the key terms “*driving*,” “*driver monitoring*,” “*driving performance*,” “*driver drowsiness*,” “*driving safety*,” “*monotony*,” and “*vehicle accidents*” is the most highly cognitive task considered in the present study for several reasons: (1) the high percentage of traffic accidents resulting from the deterioration of vigilance; (2) the high level of mental and cognitive functions required during driving; (3) the availability of advanced driving simulators or virtual reality systems that enable realistic study conditions; (4) simulated driving experiments are safer than realistic experiments [41]; and (5) contemporary EEG systems allowed for natural behavior performance [41–45].

Key terms that occur frequently and are relatively close to each other on the map can be represented in the form of clusters. Three clusters are shown in Fig 7A, including cluster 1 in red, cluster 2 in green, and cluster 3 in blue. **In cluster 1 (43 keywords)**, we observed a strong correlation between discriminating the human mental performance in a fatigue task utilizing PSD by the aid of wireless EEG device to determine the human mental state, whether “sleep,” “alert,” or “drowsiness.” “Entropy” is indicative of the nonstationary nature of EEG data. Electrocardiogram (“ECG”) has been used with EEG studies to measure the heart rate “hrv” during fatigue tasks where a significantly lower heart rate after the driving task. Fatigue task studies focus on developing fatigue “countermeasures” and “predicting” and “detecting mental states including “drowsiness” and “alertness”. The implementation of electronic alarms and devices will help to detect fatigue and thus reduce road and vehicle accidents. Furthermore, advance automatic recognition algorithms such as neural networks and machine learning algorithms, especially support vector machine (“*SVM*”), have been widely used to classify and distinguish between human mental states (e.g., alertness vs. drowsiness). In terms of EEG artifacts, “*independent component analysis*” (ICA) has been frequently applied to clean raw EEG signals. **Cluster 2 (30 keywords)** shows that that various EEG indices such as “*PSD of alpha*,” “*PSD of theta*,” “*ERD and ERS*,” “*amplitude of P300 and n*1” are affiliated with “*working memory load*,” “*attention*,” “*task difficulty*,” and “*error recognition*.” The “*n-back*” test has attracted much attention as a method to assess working memory load. Other tasks that appear in the cluster include “*driving monitoring*,” “*dual-task*,” and “*auditory*.” “*Prefrontal cortex*” is frequently observed, indicating that frontal brain regions play an active role in memory tasks. “*ERD/ERS*” (event-related desynchronization/event-related synchronization) and “*P300*” significantly change when task demands vary. **In cluster 3 (30 keywords)**, the term “*mental w*orkload” appears strongly, which is related to different “*difficulty*” levels. Several studies in the “*neuroergonomics*” area seek to develop adaptive systems (“*adaptive automation*,” “*systems*,” “*automation*,” and “*model*”) using “*EEG indexes*” and “*brain*” data for “*identifying*,” “*modeling*,” recognizing the “*risk*,” and “*operator functional state*.” Furthermore, “*neuroergonomics*” studies focused on determining the “*pattern recognition*” of “*emotions*” and “stresses” in the workplace. “*Brain-computer interface*” contributes significantly to “*neuroergonomics*” applications.

**Fig 9:**
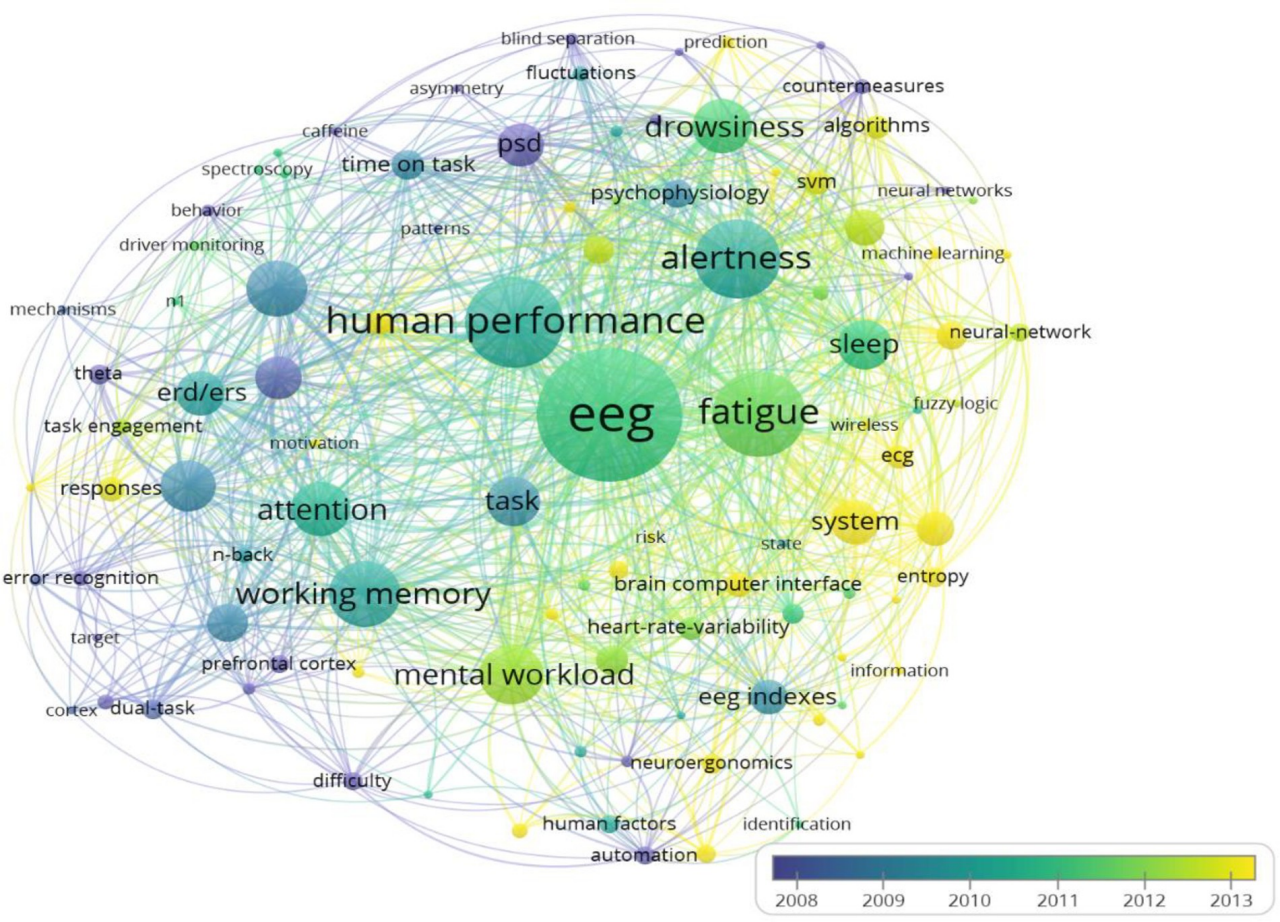
Visualization map of the evolution of keyword frequency over time.

The timeline-based visualization of different highlighted keywords is shown in Fig 9. The color of the circle indicates the time slot—the lighter the circle, the more recent the keyword. The evolution of high-frequency keywords helps researchers to determine the main research trends in cognitive work. The red dotted lines show the evolution of high-frequency keywords in recent years. The application of automatic “*algorithm*” recognition systems using “*machine learning*” (e.g., “*SVM*”) and “*neural networks*” is gaining sustainable attention for “*identification*” and “*predicting*” mental states. The application of “*brain–computer interface”* technology has recently been applied in “*neuroergonomics*” applications for the online assessment of the human mental state in cognitive work.

Applying nonlinear EEG indices, such as an “*entropy*” measure, in fatigue recognition and quantification studies is another current trend. Mobile and “*wireless*” EEG system has been used for more flexibility. The development of stress and emotion recognition systems based on brain signals is another recent area of interest.

## 4. Micro-scale analysis of the literature

In this section, we will discuss and synthesize the core data set of 143 journal articles on applications of EEG indices in cognitive work. To do so, we classified the selected articles according to the following categories of performance measures: (1) mental fatigue, (2) mental workload, (3) mental effort, (4) visual fatigue, (5) working memory load, (6) emotion and stress, and (7) error recognition. This section also describes the major findings, in line with RQ1 and RQ2.

### 4.1 The effect of mental fatigue

The assessment of mental fatigue based on neuronal data is of great interest in neuroergonomics studies [46] for evaluating occupational health and safety. Mental fatigue occurs when high levels of attention and concentration are required in a task [46–48]. For instance, monitoring modern automation systems—such as cockpit monitoring, air traffic control, seaboard navigation, military surveillance, and industrial process control—significantly increases mental fatigue [49–51]. Mental fatigue is the major reason for the deterioration of vigilance, drowsiness, sleepiness, tiredness, and loss of motivation during cognitive tasks [51–53]. Accordingly, the terms “mental fatigue,” “drowsiness,” and “sleepiness” are very closely related [25, 54]. In the current study, we categorized the factors that provoke mental fatigue into four sections: (1) drowsiness, (2) transition phase (i.e., the transition from alert to drowsy), (3) prolonged time spent on a task, and (4) task engagement.

#### 4.1.1 Mental fatigue resulting from drowsiness

Drowsiness is a decrease in the level of cognitive attention with a desire for sleep [46, 55]. Discriminating between states of drowsiness and vigilance has been conducted using linear and nonlinear EEG indices. PSD is the most frequently used EEG index to assess mental fatigue [30, 56–62]. The PSD of the alpha frequency from the occipital lobe and the PSD of theta bands from the frontal lobe appear to be the most prominent indicators in mental fatigue studies [30, 54, 63–77]. Several articles have reported increases in the PSD of theta and alpha bands during fatiguing tasks [64–68, 70, 72, 76–78]. However, Jap et al. [79] and Tanaka et al. [54] reported different results. The utilization of other frequency bands has been poorly addressed. The PSD of beta bands was shown to decline at the end of a cognitive task [54, 78–80], whereas significant increases in the frequency of delta and theta waves were observed in a few studies [30, 48, 81].

Indices derived from PSD have shown promising results in studies on mental fatigue and include average relative power, ratios of power, energy parameters, and burst. Relative power is computed by dividing the absolute power of a specific frequency band by the total spectral power of the signal and has been used in a fuzzy model for the online detection of driver drowsiness [80]. A reduction in the levels of attention and alertness is conveyed by a significant increase in the relative power of the alpha signal [66], which is consistent with results on alpha PSD. The ratios of powers such as [(α + θ)/β], [θ/α], and [α/β] appear to be reliable indicators of detecting states of vigilance [52, 79, 82–86]. A significant increase in (θ + α)/β and a decrease in θ/α or β/α are associated with increasing levels of fatigue [52, 79, 82, 86]. Another index, the alpha spindle, which is a short and narrow burst in alpha activity with a higher amplitude [87], represents a discrete event that is characterized by duration, spectral amplitude, and peak frequency [88]. The alpha spindle in the parietal and occipital regions of the brain is increased during distraction and prolonged tasks [87–91]. Sonnleitner et al. [90] combined alpha and gamma band power with alpha spindle rate and duration. An increase in the alpha spindle rate is affected by shifting from a primary task (e.g., driving) to a secondary task (e.g., an auditory task). Moreover, a higher gamma band is found only during the primary task. Furthermore, the rate and duration of the alpha spindle linearly increase with time on task (TOT), indicating the occurrence of fatigue.

Parameters of relative energy, such as wavelet package energy (WPE), have been also used to discriminate between different states of mental fatigue. It has been shown that the WPE of beta bands is significantly decreased after a mentally fatiguing arithmetic task [86], whereas the WPE of the EEG low-frequency bands was shown to significantly increase [86, 92]. The PSD or WPE of frequency bands and their respective ratios have been used as inputs in different models to predict and classify different mental states. The first countermeasure software was developed by Lal et al. [93] and used all EEG frequency bands. Subsequently, Lin et al. [74] combined an ICA with a fuzzy neural network (FNN) to predict the state of alertness, thereby generating an ICA-mixture-model-based fuzzy neural network (ICAFNN), similarly to Hsu et al. [72] and Lin et al. [94]. Other models have been developed using a least-squares multivariate linear regression model [73], kernel partial least squares decomposition [68], sparse representation [77, 95], fuzzy logic [80], and sequential discounted autoregressive algorithm [89]. Furthermore, PSD and WPE have been used to develop predictive and classification models through the application of artificial intelligence techniques, such as SVM algorithm [30, 59, 80, 81, 84, 86, 92, 96–98], artificial neural networks (ANN) [99–102], *k*-means clustering [62], Bayesian neural networks [103–105], and deep learning [106–108]. Multiple classifiers were used to identify the highest degree of accuracy for optimal prediction [63, 109, 110]. The extraction of EEG zero-crossings and integrated EEG has also been used with the aid of stationary wavelet transform (WT) as an input to a back-propagation neural network for the classification of vigilance [99].

The amplitude of some event-related potential (ERP) components and mental fatigue has been addressed by using the amplitude of P200, P300, and N100 components, and late negative difference Nd waveforms. A decrease in N100 and P200 [111] and an increase in P300 have been observed during periods of high mental fatigue [68].

Other studies have quantified EEG data using nonlinear parametric measures because EEG signals represent a nonstationary, dynamic, and nonlinear time series [112]. For instance, the level of chaos in time-series data can be measured by extracting entropy measurements, including wavelet entropy (WE), sample entropy (SE), spectrum entropy (SPEn), peak-to-peak sample entropy (PP-SampEn), approximate entropy (ApEn), peak-to-peak ApEn (PP-ApEn), Shannon’s entropy, Tsallis wavelet entropy, generalized escort-Tsallis entropy, log energy entropy, approximate entropy, Rényi’s entropy, and fuzzy entropy [26, 81, 86, 110, 113–117]. Although the use of the fractal dimension (FD) method has not been well addressed in the context of cognitive tasks, it has been applied recently in several studies on emotional states [118, 119]. Likewise, a recent study showed that a low FD value was obtained in a state of drowsiness compared with a state of arousal during a driving task [120].

EEG entropies have been used to develop automated detection systems [26, 110, 115], similar to PSD and WPE measures in the linear form. The application of EEG indices with machine learning algorithms can aid in the development of highly accurate automatic countermeasure devices for detecting, recognizing, and predicting human mental states [75, 93, 121]. An intelligent system for the detection of drowsiness was recently developed by combining PSD with nonlinear features, such as FD, SPEn, and permutation entropy [42].

#### 4.1.2 Mental fatigue resulting from transition phase

The transition phase is defined as the transition from being awake to being asleep [53]. PSD and the relative power of the five frequency bands have been used to monitor the transition phase during cognitive tasks [122, 123]. A decrease in the PSD for the alpha band was observed in the drowsiness state, whereas dominant beta activity was observed in the alert state [124]. Awais et al. [81] found an increase in the PSD of delta in the parietal and central regions, an increase in PSD of theta activity in the parietal region, and an increase in PSD of alpha in parietal, central, and occipital regions during the transition phase. However, inconsistent EEG results have been observed, demonstrating no change in theta power [57] and variation in alpha power [125]. The relative power of the alpha band was shown to increase significantly in the parietal [80] and occipital [81] regions during the transition phase. Nguyen et al. [126] reported an increase in the relative level of power in lower-frequency bands and a decrease in the relative level of power in high-frequency bands. Sample entropy has also proven very useful in detecting the transition phase in terms of the time domain, indicating a reduction in EEG signals in the occipital and parietal regions during the shift from alertness to drowsiness [81]. Similar results have been shown for Shannon’s entropy [127].

#### 4.1.3 Mental fatigue resulting from prolonged time spent on a task

The state of fatigue is associated with the length of time spent on a task (TOT) [128]. In general, as TOT increases, human performance deteriorates, and mental fatigue begins to occur. A significant increase in the PSD of alpha, theta, and (alpha + theta) bands in the occipital, medial, and frontal regions is observed during fatigue over long periods [47, 65, 70, 78]. In contrast, significant increases in the PSD of beta bands have been observed in the occipital [60, 128] and lateral frontal regions of the brain [70], which contradicts other major findings in the literature [65, 78]. Lafrance and Dumont [69] explained this conflict by noting that an increase in the effort to stay alert to accomplish a given task results in an increase in the PSD of beta bands. Trejo et al. [68] reported that TOT significantly affected the amplitude of P200 but did not affect either the amplitude or latency of N100, P200, or P300. However, a slight increase in the latency of P300 and a reduction in the amplitude of P300 was observed by Zhao et al. [78]. In addition, the amplitude of N1 has been shown to decrease with TOT [70, 129, 130].

#### 4.1.4 Mental fatigue resulting from task engagement

Task engagement is a positive, excited state that is influenced by workload state, TOT, motivation, and emotions [51, 131, 132]. Assessing the deterioration of attention that results from task engagement using EEG indices has attracted the attention of many researchers. The observation that the amplitude of P300 attenuates during task engagement has been linked to the presence of mental fatigue [50, 129, 131, 133–135]. For example, a decrease in the amplitude of N1 and an increase in the amplitude of N2 have been reported during task engagement [70, 130, 131, 136]. Furthermore, reductions in job motivation and performance are marked by a decrease in error-related negativity/error negativity (ERN/NE), the amplitude of N2, and the amplitude of contingent negative variation [131].

The EEG-engagement index β/(θ + α), proposed by Prinzel et al. [137], is defined as “the ratio between beta power and the sum of theta and alpha power associated with certain EEG measurement channels” [138] and can be used to detect the deterioration of task engagement [139]. A reduction in the engagement index during vigilance tasks demonstrates the deterioration of task engagement over time [140–142]. There is a direct correlation between the EEG-engagement index and task load [140], making the index very effective in quantifying the state of the workload.

### 4.2 The effect of mental workload

As a multidimensional construct, the mental workload has been generally defined in terms of the resources available to meet a task’s demands [143–153]. Not only does excessively high workload reduce human performance, but a workload that is too low reduces the motivation and interest of the operator for the job [154]. In high workload scenarios, resources that are allocated to perception are depleted, resulting in deafness to auditory alerts, neglect of all incoming information, a slowing of the decision-making process, and a deterioration in vigilance. Therefore, a moderate workload is necessary to maintain a safe and productive working environment. Taking human brain data into account should aid in the precise and continuous evaluation of the mental state and effort of the operator (see [155] for review). Many studies have focused on discriminating between high, moderate, and low workloads of cognitive tasks with the aid of EEG indices [156, 157].

The PSD of the frontal and occipital theta bands and the PSD of parietal alpha bands have been used extensively to assess mental workload. A reduction in the PSD of the parietal alpha bands and an increase in the PSD of the frontal theta bands have been observed when task difficulty increases [46, 154, 156–164]. Similar results have also been observed when using WT of EEG signals [156]. Another parameter derived from EEG signal power is ERD/ERS, where the reduction in power is called ERD [165] and its increase is referred to as ERS [166]. Theta ERS increases linearly with an increase in task demand [167], and a significant correlation has been observed in a wide range of cognitive tasks between the upper and lower alpha ERD when task demands increase [168]. The task load index (TLI) is defined as the ratio of the mean frontal midline theta energy to the mean parietal alpha energy. TLI has been shown to increase over time during different cognitive tasks [141, 169–171]. The engagement index β/(θ + α) is highly recommended for designing adaptive systems [172]. The purpose of an adaptive system is to enhance mental engagement and situational awareness by maintaining a moderate workload. When the value of the engagement index increases, the system switches to automatic mode, resulting in a decrease in the EEG-engagement index. In contrast, when the engagement index value decreases, the task is switched to manual mode, which in turn increases the workload and subsequently increases the EEG index [135, 137, 139, 172–175].

Recent studies have reported that high task engagement with a very high visual load reduces auditory processing. This novel phenomenon is known as “inattentional deafness: the inability of the auditory stimulus to reach consciousness” [176]. The difficulty of the task consumes most of the attention, leaving few or no resources available to process any other information. Traffic management studies aim to enhance the brain’s response to audible warning alarms and to design intelligence alarm signals by studying this phenomenon [176–180]. A higher engagement index has been observed in the fronto-central and parietal areas during complex piloting tasks [179]. Dehais et al. [181] described a number of suboptimal neurocognitive states that significantly reduce human performance, such as perseveration, effort withdrawal, mind wandering, and inattentional blindness and deafness.

The amplitude of some ERP components (e.g., P3, P2, N1, N2, late positive potential [LPP], mismatch negativity [MMN], early slow-wave component [SW1], and late slow-wave component [SW2]) decrease as the workload increases. In contrast, the latency of the ERP component increases as the workload increases [176, 182–187]. Other studies have combined PSD and the amplitude of ERP components, mainly P300 [169, 182, 188]. These studies have demonstrated a decrease in P300 amplitude and the PSD of alpha bands but an increase in the PSD of theta bands when workload increases. In comparing spectral markers and spatially filtered ERPs, Roy et al. [55] concluded that the spatially filtered ERP method is more accurate and more efficient at discriminating workload levels than using the spectral domain. An early study by Murata et al. [189] applied nonlinear parameters, including the largest Lyapunov exponents (L1), FD, and attractor plot, to the quantification of workload. That study concluded that only FD could be used to evaluate human mental workload. Several computational intelligence algorithms have been used to classify and detect mental workload levels, such as SVM [72, 138, 190–192], ANN [193–195], and random forest (RF) [196].

### 4.3 The effect of mental effort

Mental effort is defined as “the cognitive capacity that is allocated to accommodate the demands imposed by the task” [197], where a high level of effort is known as “strain” [170]. Dasari et al. [128] reported a significant correlation between the PSD of the frontal theta and alpha bands and mental effort. However, Sauseng et al. [198] observed a reduction in the PSD of upper alpha activity during tasks that required a high level of mental effort. The suppression of alpha activity provides information regarding the mental effort in arithmetic tasks [154].

### 4.4 The effect of visual fatigue

Visual fatigue is developed stress in the eyes when a task requires continuous eye focus for long periods (i.e., generally from 6 to 9 hours). Difficulty focusing, headaches, eye aches, eye soreness, and blurred vision [199] are visual fatigue symptoms that degrade performance at work. A linear relationship exists between the level of visual attention and the rate of error [167, 200]. Visual fatigue is characterized by an increase in the PSD of theta and alpha bands in the occipital region [201]. Furthermore, an increase in the [(α + θ)/β] ratio confirms the gradual decrease in attention during prolonged tasks. However, contrasting results were observed by Chen et al. [202]. A reduction in the alpha and beta bands and an increase in the delta band and the ratio of powers for the indices $\frac{\theta +\alpha }{\beta },\frac{\theta }{\beta }$, and $\frac{\theta +\alpha }{\beta +\alpha }$ were found, with no change in the theta band.

In a 3-dimensional (3D) oddball paradigm, Li et al. [203] found that the PSD of the beta frequency and the peak difference in the ERP component, mainly P700, are reliable indices for assessing 3D visual fatigue. The beta power is higher in 3D visual tasks than in 2-dimensional (2D) visual tasks. Furthermore, a delay in the P700 component has been investigated in a 3D task rather than in 2D. Cao et al. [52] combined PSD and the amplitude of the steady-state visual to characterize EEG data during a visual fatigue task. A strong correlation was observed between the gravity of the PSD and power spectral entropy during a visually fatiguing task [204], whereas a reduction in the gravity of the PSD and power spectral entropy was found during a long visual task. Wiyor et al. [199] developed a predictable detection model for estimating and classifying visual fatigue using ANN.

### 4.5 The effect of working memory

Working memory is the ability to maintain and manipulate information for a certain period of time [205]. Several factors significantly affect human working memory, including interfering stimuli, cognitive load, task practice, and aging [206]. In the current study, we focused on reviewing the effects of cognitive load and task practice on working memory load. The PSD of the theta and alpha bands is very sensitive to increases in working memory load. An increase in the frontal theta bands and a decrease in the parietal alpha bands have been observed in several studies [15, 161, 171, 198, 207–209]. During the retention of memory, the PSD of alpha [210] and theta [207] activity increased with increasing levels of working memory load. Consequently, Jensen et al. [211] highlighted the importance of fast cortical oscillatory activity, mainly gamma activity, in attention and working memory during complex tasks.

ERP components, such as the amplitude of early P1 and P3a and the amplitude and latency of P300, have been used to quantify the human attention state during working memory tasks. For example, during high working memory load, a reduction in the amplitude of P300 is found in the parietal and frontal regions [212–215]. Combining PSD with ERP components can be used to reflect the mental state in working memory tasks. An increase in the PSD of the theta band, a decrease in the PSD of both alpha and beta bands, and a decrease in the amplitude of P300 have been observed during two different working memory tasks [216]. Working memory load was examined according to the power of ERD/ERS from 4 to 12 Hz by Krause [217]. A greater cognitive load was reflected by an increase in the ERD of 10 to 12 Hz frequency bands.

### 4.6 The effect of emotion and stress

Emotions play an important role in overall human performance because they significantly affect cognitive functioning, decision-making, and individual performance [132, 218]. Hence, understanding human feelings and emotions in the workplace is essential in providing safe working environments, especially in high-risk work such as maritime, aviation, and site construction [192, 219]. Stress results from emotional pressure [220]; consequently, emotions and stress are tightly correlated [221]. Emotional recognition models have been developed by using EEG indices as input parameters. A study by Blaiech et al. [222] used the PSDs of the five frequency bands as input variables to fuzzy logic-based analysis methods. Moreover, Calibo et al. [223] used the root mean square (RMS) voltage for theta, alpha, and beta bands as an input to logistic regression and a *k*-nearest neighbor classifier algorithm to classify “stressed” and “nonstressed” conditions. Another study developed a monitoring system, called “CogniMeter,” to recognize stress, emotions, and workload levels in a maritime simulator and in air traffic control [221]. Results revealed highly accurate emotional recognition models that can classify and recognize human emotional state, workload, and stress in real time. The use of chaos methods, mainly FD, is a highly effective method for recognizing and quantifying human states of emotion [224, 225]. A reduction in the FD value has been shown to correspond to negative emotions [224]. A positive correlation between mental workload and stress was observed when combining PSD with FD indices to recognize different emotional states and mental workload and was shown to successfully reflect human emotions [190, 225, 226].

### 4.7 The effect of error recognition

Detecting and analyzing human error is crucial to enhancing the performance of the human-machine system and preventing incidents in the workplace. ERP components, mainly ERN, provide useful information from brain signals associated with different types of errors [227]. Negativity in the ERP is shown when performing an error and detecting errors committed by others [228]. Kim et al. [229] studied the PSD to minimize risk in nuclear power plants. The absolute power of alpha increased when participants answered questions correctly, showing more relaxed and stable emotions when there was no human error. In contrast, the absolute power of the beta band, the absolute power of the gamma band, the relative power of the theta band, and the (θ/α) ratio significantly increased when wrong answers were given, indicating the occurrence of stress and fatigue and a decrease in relaxation. During the low-error period, an increase in the beta and gamma tonic power spectra were found [200]. https://books.google.com/books?id=G1hiDwAAQBAJ&pg=PA145&lpg=PA145&dq=neuroergonomics+in+nuclear+power+plant&source=bl&ots=ze9a3RcYvW&sig=ACfU3U01Bvi-SVEOPu84JXdqbpX2tg7fHg&hl=en&sa=X&ved=2ahUKEwjAkLiJ5tHiAhWtT98KHX-7AXQQ6AEwFnoECAkQAQ-v=onepage&q=neuroergonomics%20in%20nuclear%20power%20plant&f=false.

## 5. Summary statistics and major findings

**Fig 10:**
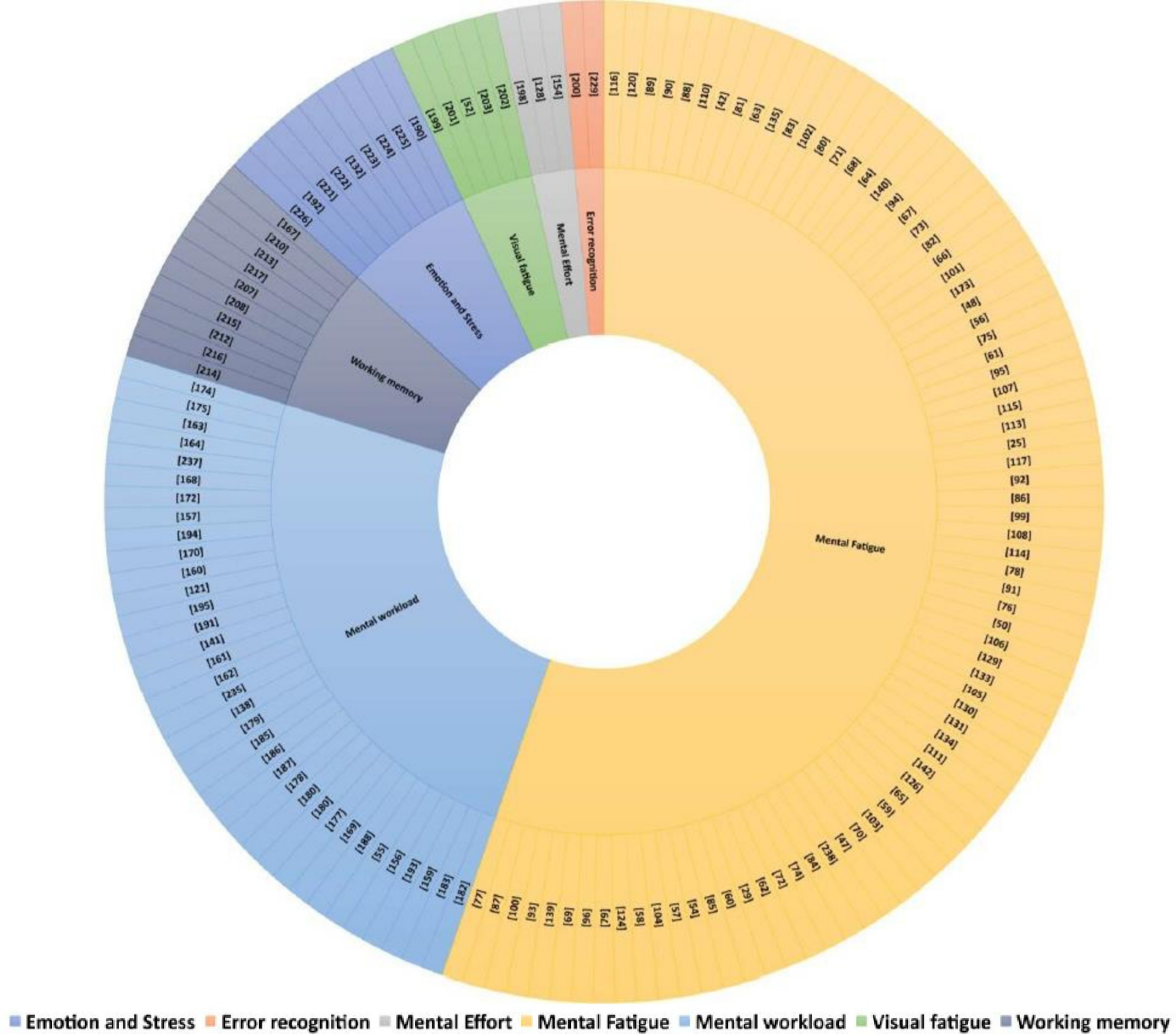
The distribution of 143 articles by performance measurements.

In the previous section, we discussed seven performance measurements for applications of EEG indices. An abstract visualization of all reviewed articles showing the distribution of performance measurements is given in **Fig 10**.

According to the figure, studies that have applied EEG indices in cognitive work have primarily focused on analyzing the effect of mental fatigue, followed by mental workload.

**Fig 11:**
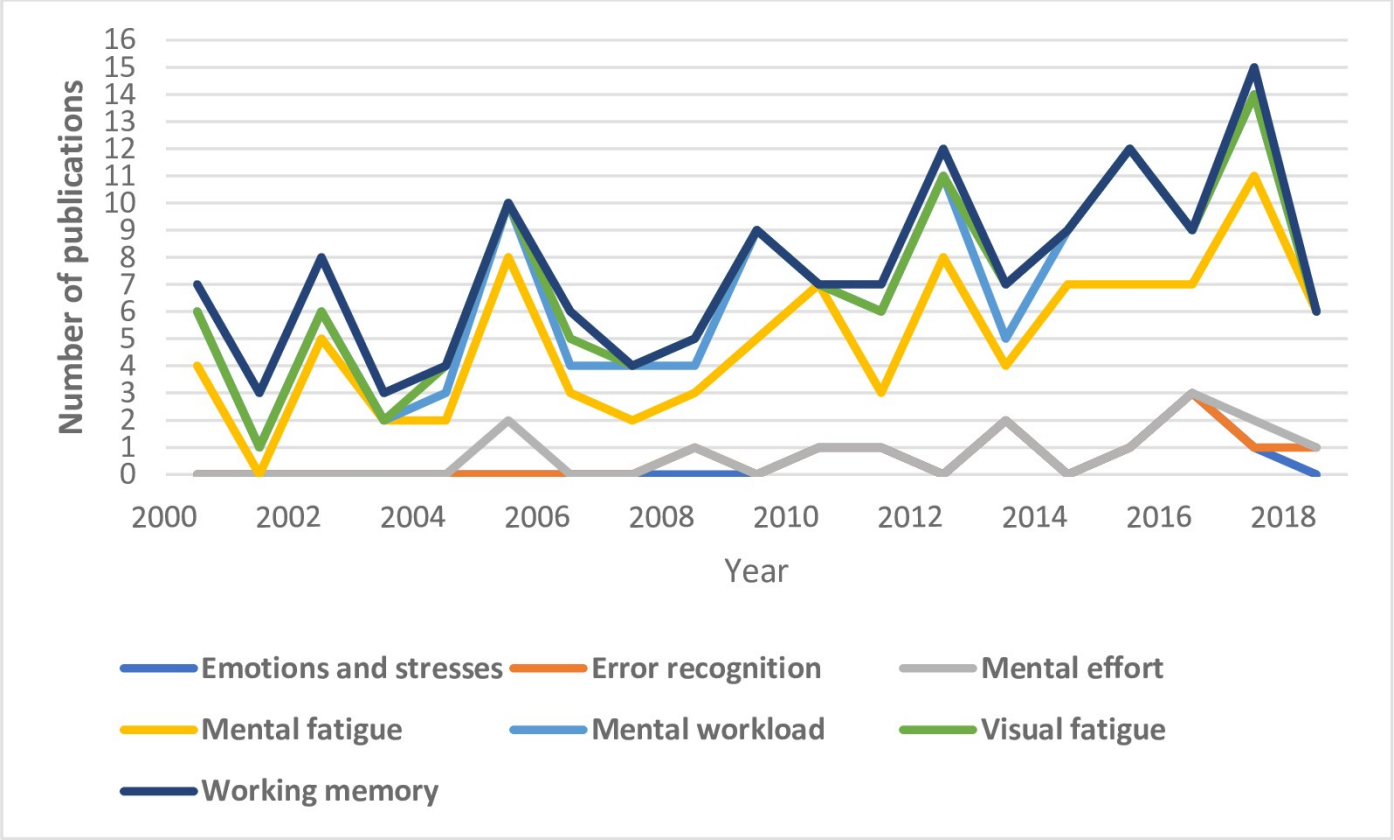
Temporal trends of the number of publications on each of the seven themes.

The assessment of EEG signals during periods of stress and emotion, visual fatigue, and mental effort has been less frequently addressed. In terms of temporal evolution of performance measures using EEG indices, mental fatigue and mental workload have attracted the most attention during the predefined time span **Fig 11**. Traditional linear analysis methods have dominated in analyzing EEG signals, whereas 6% of the reviewed studies applied nonlinear analysis methods, and 4% of the reviewed articles applied a combination of linear and nonlinear methods. The evidence indicates that PSD is the most frequently used EEG index, followed by the amplitude and latency of some ERP components.

The driving task has constituted the majority of published neuroergonomics applications, followed by tracking and monitoring human attention for simple cognitive tasks (e.g., matching or detecting color changes) and complex cognitive tasks (e.g., aircraft control systems, air management systems, power plants, cement work, traffic control, and maritime activities). For cleaning EEG signals from artifacts, filtering, manual visual inspection, ICA, WT, autoregressive models, artifact substance reconstruction, principal component analysis (PCA), and subtraction methods have commonly been applied. Various methods have been applied to extract the features of nonstationary EEG signals, primarily including Fourier (FT) transforms. Kumar et al. [230] suggested applying a multi-base scale such as WT, because it extracts both time and frequency domains. “The accuracy becomes higher when FFT is replaced by wavelet packet energy” as mentioned by Zhao et al. [92]. Rabbi et al. [35] reported that the WT technique and short-time Fourier transform (STFT) are more efficient than the standard FT. Following feature extraction, feature selection is performed to select the most descriptive features (i.e., data mining step). The features are classified according to Gaussian linear or quadratic classifiers, regression methods [73], linear PCA [92], Fisher linear discriminant analysis (FLDA) [126, 231], PCA with FLDA [58, 84], or ICA [72]. Recently, sophisticated algorithms and intelligence techniques, including machine learning algorithms, have been used to classify EEG features, especially when more than two states must be classified [232]. The most prevalent machine learning algorithm identified in the present study was the SVM method. All relevant statistical data are summarized in Table 1.

**Table 1:** A comprehensive summary of the findings from the review.

	Cognitive activity-based EEG studies			
Performance measurements	EEG indices	Cognitive tasks	Methods of artifact removal	Methods of feature extraction
• Mental fatigue 57% • Mental workload 22% • Working memory load 7% • Emotional stressors 4% • Visual fatigue 3% • Combination 6% • Human error 1%	PSD 59%	• Vehicle driving (n = 55) • Tracking and monitoring (n = 41) • N back task (n = 8) • Arithmetic task (n = 6) • Multiple cognitive tasks (n = 6) • MATB (n = 5) • Auditory task (n = 4) • Working memory task (n = 5) • Stroop test (n = 3) • Sleep latency test (n = 3) • Sternberg task (n = 3) • Gaming (n = 3) • Simon task (n = 1)	• Filters (n = 69) • Manual rejection (n = 45) • ICA (n = 31) • WT (n = 3) • AR (n = 3) • ASR (n = 2) • PCA (n = 2) • Subtracting method (n = 2)	• FFT (n = 56) • Averaging time-locked (n = 16) • WT (n = 9) • Welch’s method (n = 7) • STFT (n = 7) • Different software (n = 6) • Higuchi and Grassberger algorithm (n = 5) • Entropy-based (n = 5) • ICA/PCA (n = 4) • AR (n = 3) • WPE (n = 2)
	ERP 15%			
	PSD and ERP 9%			
	Linear and nonlinear 4%			
	WPE 3%			
	Entropies 3%			
	Alpha spindle 3%			
	FD 2%			
	FD and entropies 1%			
	FD and DWT 1%			
	Temporal and spatial covariance 1%			
	Sparse representation 1%			

Power spectrum density [PSD], event related potentials [ERP], wavelet package energy [WPE], discrete wavelet transformed [DWT], independent component analysis [ICA], wavelet transformed [WT], Artifact Subspace Reconstruction [ASR], principal component analysis [KPCA], fast Fourier transform [FFT], short-time Fourier transform [STFT], wavelet package energy [WPE].

## 6. Research gaps and future directions

In this systematic review, we explored the feasibility of using EEG indices to quantify human performance during various cognitive tasks. Our results reveal the growing interest in applying traditional linear EEG indices to investigate cognitive tasks. Nonlinear analyses, mainly FD, have recently gained considerable attention as a means to assess emotional states [118, 190, 224–226]. Although substantial advances have been made in the use of EEG indices to quantify human performance during work, several challenges remain to be addressed regarding the nonstationary nature of EEG signals [112].

Therefore, computational methods, dynamic nonlinear methods, nonlinear time series-based analyses, and rigorous statistical analyses to convey the aperiodic nature of the EEG signals are promising areas for the quantification of human performance during work (i.e., neuroergonomics). There is growing interest in studying brain function as a complex network based on modern network science [233]. Furthermore, a queuing network-based computational neuroergonomic architecture [234] is a potential approach to the development of recognition and adaptive systems that can make correct decisions in a short period of time [235]. Although some researchers have predicted current human states using machine learning algorithms, the successful application of such algorithms has been limited [236]. Another shortcoming is the lack of designs with ecological validity. Few studies have characterized human brain signals during real application [114]. The majority of cognitive experiments described in the current study have been limited to laboratory conditions [42, 237, 238]. The assessment of multiple cognitive tasks that reflect real-life situations should be addressed in future studies, as they accurately reflect real-life situations. The effects of mental state on physical performance have not yet been thoroughly investigated. Finally, considerable additional research are needed for better classifying the momentary cognitive fatigue states [239].

## 7. Conclusions

This systematic review explored the applications of EEG indices to quantify human cognitive performance based on a bibliometric analysis of selected papers published between 2000 and 20018. To the best of our knowledge, this is the first study to apply bibliometrics analysis at the macro and micro scales for the analysis of the EEG indices in cognitive work. Based on the evaluation of 143 studies extracted from Web of Science, we revealed considerable changes in EEG indices during specific performance measurements, including mental fatigue, mental workload, working memory, emotional and stresses, visual fatigue, and error recognition. We also described mapping analysis for the reviewed papers using a bibliographic coupling, co-citation, affiliation, and co-occurrence of keywords.

The presented review allowed a comprehensive analysis of the leading research trends relevant to cognitive neuroergonomics. An increasing trend for publication in this area was observed over the last ten years, with the highest number of publications in 2017. Most studies applied EEG power spectral density as linear methods to evaluate human cognitive performance. Subsequently, the FFT has been used to extract the power spectrum. One limitation is that the choice of the optimal EEG index remains unclear. Evaluating an individual’s mental states, especially while driving a vehicle, has been most frequently studied, closely followed by tracking and monitoring tasks and various working memory tasks. Although several artifact removal methods have been used, the application of filters in addition to manual visual inspection was prevalent. Future research should focus on applying computational methods that consider the dynamic and non-stationary nature of EEG data. Such an approach can facilitate the development of fatigue recognition systems and automatic adaptive systems. Finally, to overcome the current limitations to characterizing and predicting human performance using EEG data, machine learning algorithms should be used.

## Supporting information

S1 Appendix (DOCX)
