# Supplementary material for: Applications of EEG indices for the quantification of human cognitive performance: A systematic review and bibliometric analysis
Source: PLoS One. 2020 Dec 4;15(12):e0242857. doi: 10.1371/journal.pone.0242857 (PMC7717519; doi:10.1371/journal.pone.0242857)
Supplement: S1 Appendix — (DOCX) [file pone.0242857.s001.docx]

A summary of papers related to cognitive tasks reviewed by present study (artificial neural network [ANN], autoregressive [AR], continuous wavelet transform [CWT], electroencephalography [EEG], electrocardiogram [ECG], electromyography [EMG], electrooculogram [EOG], event-related desynchronization [ERD], event related potentials [ERP], event-related synchronization [ERS], event-related spectral perturbation [ERSP], convolutional neural network [CNN], dynamic Bayesian network [DBN], discrete wavelet transformed [DWT], fast Fourier transform [FFT], independent component analysis [ICA], k-n neighbor [k-NN], linear discriminant analysis [LDA], least-squares SVM [LSSVM], Magnetoencephalography [MEG], multi-layer perceptron neural network [MLPNN], not mentioned [NM], power spectrum density [PSD], permutation entropy [PmEn], radial basis function neural network [RBFNN], root mean square [RMS], task load index [TLI], virtual reality [VR], wavelet package energy [WPE], wavelet transformed [WT], support vector machine [SVM], SVM with kernel principal component analysis [SVM- KPCA], stepwise linear discriminant analysis [SWLDA], self-organizing neural fuzzy inference network [SONFIN], spectral entropy [SPEn]).

| **Study #** | **Reference**  **#** | **EEG indices** | **Physiological measurement** | **Performance measurements** | **Applications in the Cognitive Task** | **Artifact removal method** | **Feature extraction,  selection, & classification** |
| --- | --- | --- | --- | --- | --- | --- | --- |
| 1 | [174] | PSD of  θ, α, β, engagement index  $\frac{\beta}{\alpha+\theta}$ & $\frac{\beta}{\alpha}$ | 22 channel EEG | Engagement level | Tracking task of the Multiple-Attribute Task battery (MATLAB) | Manually rejecting | FFT for PSD |
| 2 | [175] | Engagement index $\frac{\beta}{\alpha+\theta}$ | Four-channel EEG | Engagement level | Tracking task of the Multiple-Attribute Task battery (MATLAB) | Manually rejecting | FFT for PSD |
| 3 | [217] | PSD of the  θ, upper α lower α, ERD & ERS | 20 channel EEG | Working memory load | Visual sequential letter task (n-back task) | Filters | NM |
| 4 | [69] | PSD of δ, θ, α & β | Eight-channel EEG | Mental fatigue (alertness versus sleep) | Sleep latency test | Manually rejecting | FFT for PSD |
| 5 | [61] | PSD of θ, α & β | Four-channel EEG | Mental fatigue (Vigilance) | Visuomotor compensatory tracking task | Manually rejecting | FFT for PSD using Hanning-windowed |
| 6 | [67] | Relative PSD of α | Four-channel EEG & EOG | Mental fatigue (Attention) | Simulated driving | Bandpass Filter | PSD was carried out using the ‘DAOS’ environment |
| 7 | [87] | PSD of α and spindle | EEG | Mental fatigue  (Vigilance) | Simulated driving | NM | FFT for PSD |
| 8 | [164] | Log PSD of θ & α | 28 channels  EEG | Workload  (task difficulty) | MATB visual display | Adaptive filters &  visually inspected | NM |
| 9 | [56] | PSD of δ, θ, α & β | 25 channel EEG | Mental fatigue (Sleep deprivation) | Aircraft flight simulation task | Low,  high & notch filter | FFT for PSD |
| 10 | [207] | PSD of θ | EEG and MEG | Working memory load | Sternberg task. | NM | Wavelet-based time-frequency analysis |
| 11 | [48] | PSD of δ, θ, α & β | 24 channel EEG& EOG | Mental fatigue (transition phase from alert to fatigue) | Simulated driving | High-pass filter. | FFT for PSD and ANOVA was used for discriminating  fatigue phases |
| 12 | [173] | PSD of θ, α, β, &  engagement index  $\frac{\beta}{\alpha+\theta}$ | 22 channel EEG | Mental fatigue (vigilance detection) | Monitoring task | visual inspection | FFT for PSD |
| 13 | [100] | PSD of α & β | 14 EEG & EOG | Mental fatigue (alert and drowsy) | Simulated driving | Bandpass filtered and visual inspection | An automatically neural network for features extract |
| 14 | [158] | Relative PSD of δ, θ, α & β | 20 channel EEG, ECG EOG &EDA | Mental workload | Piloting | Manscan software package | NM |
| 15 | [93] | PSD of δ, θ, α & β | EEG & EOG | Mental fatigue | Simulated driving | Filters and adaptive motion– artifact reduction | FFT for PSD |
| 16 | [238] | PSD of θ, α & β | 6 electrodes EEG & EMG | Cognitive workload | Warship Commander Task and memory Task | Filters and automatic detection | Stepwise linear regressions for discrimination  “B alert “ |
| 17 | [201] | Absolute SPD for θ, α, β, &  $\frac{\alpha+\theta}{\beta}$ | Four electrodes EEG | Visual fatigue and loss of vigilance | Simulated driving | NM | NM |
| 18 | [139] | PSD of θ, α & β | Four sites EEG | Mental fatigue (vigilance) | Target detection and monitoring task | Filters | FFT for PSD |
| 19 | [101] | PSD of δ, θ, α & β | Eight-channel EEG | Mental fatigue (alert, drowsy & sleep) | Simulated driving | Bandpass filter | DWT |
| 20 | [73] | Log subband power ICA/EEG spectrum for five frequency bands | 33-channel EEG, EOG & ECG | Mental fatigue and error recognition (Drowsiness & driving error) | Simulated driving | ICA | ICA |
| 21 | [82] | Basic index the PSD of θ, α & β; the ratio indices  $\frac{\theta}{\alpha}$ , $\frac{\beta}{\alpha} and \frac{\alpha+\theta}{\beta}$; and the burst for θ & α | Eight channels EEG | Mental fatigue (Drowsiness & sleepiness) | Simulated driving | ICA and correlation density | The preprocessed data were separated into bands through bandpass filters |
| 22 | [168] | ERD for α band | 27 EEG & EOG | Mental Workload (difficulty of the task) | Multiple cognitive tasks  1.Letter matching task 2. Triple number test 3.speed of information processing task (counting) 4.working memory task | Filters | Averaged over all recording positions |
| 23 | [65] | PSD of (θ + α) | Four electrodes EEG | Mental fatigue (alertness and sleep deprivation) | Simulated driving | Visual inspection | Power spectra by Brain Vision Analyzer |
| 24 | [154] | PSD of α | EEG, EOG & ECG | Mental workload | Arithmetic task | Band-pass filter, visually inspected and subtracting the data segmentation | FFT for PSD |
| 25 | [198] | PSD of upper θ & α  ERS/ERD | 30 channels  EEG, EOG | Mental Effort | visuospatial  working memory task. | NM | NM |
| 26 | [172] | The PSD of θ, α, β &  Engagement index  $\frac{\beta}{\alpha+\theta}$ | EEG | Mental workload | MATB | Artifact rejection subroutine | FFT for PSD |
| 27 | [74] | Log PSD | 33 channels EEG | Mental fatigue (drowsiness) | VR  driving | ICA | FFT for PSD then adaptive feature selection mechanism and ICAFNN |
| 28 | [94] | PSD of α | 34 channels EEG, two-channel EOG, and two-channel ECG | Mental fatigue (drowsiness) | VR driving | ICA | ICA |
| 29 | [140] | Absolute and relative PSD | Six bipolar electrodes EEG | Task Engagement, mental workload, and working memory | Forward/backward digit span, grid recall, mental arithmetic, and trails. | Filters and  wavelets transformation | FFT for PSD  For classifying workload, the quadratic and linear discriminant functional analysis |
| 30 | [157] | PSD of δ, θ, α, β, & γ | 32 channels EEG and ECG | Cognitive Workload | Flight simulator with a secondary auditory task | Filters | FFT and Welch method for PSD |
| 31 | [76] | PSD of θ & α | 30 channels EEG | Mental fatigue | Mental arithmetic task | Manually rejected, low pass filtered then automated artifact rejection | Welch’s periodogram |
| 32 | [194] | PSD of δ, θ, α, β & γ | Five-channel EEG, EOG, and ECG | Cognitive workload | Aerial vehicle task | Adaptive filter | FFT for PSD ANN for discrimination between high and low workload |
| 33 | [200] | PSD of δ, θ, α & β,  ERSP methods and a full-spectrum extension of ERD and ERS measures, | 70 channel EEG | Mental fatigue (drowsiness) | Continuous compensatory tracking task | ICA using EEGLAB | FFT for PSD by Hann window |
| 34 | [71] | Log PSD of α & θ | 28 channel EEG | Mental fatigue (drowsiness) | VR-based driving simulator | lowpass filter | FFT for PSD using Hanning windows and the alert model using Mahalanobis distance. |
| 35 | [96] | PSD of δ, θ, α & β | 19 channel EEG | Mental fatigue | Auditory vigilance task | visual inspection. | FFT for PSD by Hann window and SVM for classification |
| 36 | [170] | PSD analysis  for δ, θ, α & β, and TLI | 32 EEG,  ECG, EOG & HCV | Mental workload | Process control task (  Cabin Air Management System) | Filters and  visual inspection | FFT by 10% Hanning window and normalized to get PSD |
| 37 | [79] | PSD of  δ, θ, α & β; the ratio of  $\frac{\theta}{\beta}$ , $\frac{\alpha}{\beta}' \frac{\alpha+\theta}{\beta}$ &$\frac{\alpha+\theta}{\beta+\alpha}$ | 30 channels EEG & EOG | Mental Fatigue (Alert state) | Simulated driving | Identify blink artifacts by the aid of EOG data | FFT using Lab VIEW programming language for extracting four frequency components |
| 38 | [193] | PSD of δ, θ, α & β | 19 electrode EEG, ECG & EOG | Mental workload (task difficulty) | Uninhabited air vehicle simulator. | Corrected using the routine in the ManScan (SAM Technology) data  analysis package and visually inspected | FFT for PSD  ANN as classifier |
| 39 | [124] | PSD δ, θ, α & β | 32 channel EEG & EOG | Mental Fatigue (transition phase from alertness to drowsiness) | Simulated driving | Visual inspection and bandpass filter | FFT with a Hann window  SVM as a classifier from drowsy to alert |
| 40 | [75] | PSD θ & α | Three EEG | Mental Fatigue (drowsy) | Simulated driving | Low pass filter | FFT for PSD |
| 41 | [58] | Spectral power for  δ, θ, α & β; the ratio of  $\frac{\theta}{\beta}$ , $\frac{\alpha}{\beta}' \frac{\alpha+\theta}{\beta}$ &$\frac{\alpha+\theta}{\beta+\alpha}$ | 62 channels EEG and 2 channel EOG | Mental fatigue (vigilance) | sleep task | Manually removed and low-pass filter along with PCA algorithm | The fisher score algorithm for features selection.  SVM for to vigilance classification. |
| 42 | [104] | PSD of α | ECG and EEG | Mental Fatigue | Simulated driving | Bandpass filter and ICA | FFT for PSD  DBN for classifying |
| 43 | [132] | PSD of θ, α & β | 19 channels  EEG | Emotion in task engagement | Gaming paradigm. | Filters and visually inspection | FFT for PSD |
| 44 | [161] | PSD of θ & α | 32 EEG & ECG | Mental Workload | The simulated driving task with lane change and  working memory load mainly N-back task. | ICA using  EEGLAB | FFT for PSD |
| 45 | [121] | PSD | 64 EEG and  EMG | Mental workload | Simulated driving | Filters | Classifying using regularized linear discriminant analysis |
| 46 | [195] | Normalized log PSD of δ, θ, α, β & γ | 19 channel EEG, ECG, and EOG | Mental workload (task difficulty) | MATB | Filters | Extraction is NM Comparing the best classifier ANN, SVM, and LDA. |
| 47 | [57] | PSD of θ, α & β | 32 EEG & EOG | Mental fatigue | Simulated driving | ICA with EEGLAB toolbox,  second-order blind source separation &  canonical correlation analysis | FFT using a Hanning window  multivariate analysis of variance for discriminating |
| 48 | [63] | PSD of θ & α | 33 channels EEG & 2 channels ECG | Mental Fatigue (drowsy) | VR Based Dynamic Driving Simulator | ICA using  EEGLAB Toolbox | FFT by Hanning windows then zero-padding  Prediction models using SVM, MLPNN, RBFNN & SONFIN |
| 49 | [80] | The average relative PSD in α and β band | Four channels EEG & EOG | Mental Fatigue (drowsy) | Simulated driving | variance comparison test | STFT for PSD extraction  Fuzzy logic for system for detection |
| 50 | [208] | PSD in  4–8 Hz, and 8–25 Hz | 64 channels EEG | Working  memory load | N-back task | Filtered, Fast ICA and visual inspection | WT |
| 51 | [54] | PSD of δ, θ, α & β; the ratio of $\frac{\theta}{\beta}$ , $\frac{\theta}{\alpha}$ | Eleven electrodes EEG, ECG& EOG | Mental fatigue | 0-back or 2-back test  N back test | visually inspected | FFT for PSD |
| 52 | [85] | Mean PSD  θ, α, β and six types of ratio indices  $\frac{\theta}{\beta} ,\frac{\alpha}{\beta} , \frac{\alpha}{\theta} ,\frac{\theta}{\alpha+\beta}$  $\frac{\theta+\alpha}{\beta} , \frac{\theta+\alpha}{\beta+\alpha}$ | 16 channel EEG | Mental Fatigue | Simulated driving | ICA, with an auto-regression | FFT for PSD |
| 53 | [222] | PSD of δ, θ, α, β & δ | EEG | Emotion | Emotion experience by created scenarios | Bandpass filter | FFT and fuzzy logic techniques as a classifier |
| 54 | [202] | PSD of δ, θ, α & β; the ratio of $\frac{\theta+\alpha}{\beta}$, $\frac{\theta}{\beta} ,\frac{\alpha}{\beta}$ & $\frac{\theta+\alpha}{\beta+\alpha}$ | 62 channels EEG & EOG | Visual fatigue | watching 2D or 3D television. | Manually eliminated | WT for feature extraction |
| 55 | [60] | PSD of θ, α & β | 128 channels EEG | Mental fatigue | Air traffic control | ICA in EEGLAB and bandpass filter | FFT with Hanning window |
| 56 | [199] | PSD of δ, θ, α, β & γ | 16 channel EEG | Visual fatigue | Visual display task | Filters | FFT for PSD  ANN as classifier |
| 57 | [191] | TLI  the ratio, and PSD of θ & α | EEG and HR | Cognitive Workload | Monitoring task | NM | LSSVM and weighted  LSSVM as classifier |
| 58 | [30] | The average log band power for δ, θ, α & β, and PSD of δ, θ, α & β | Four channels EEG | Vigilance prediction | VR driving task | band-pass filter | FFT using Welch’s method and zero paddings for extracting PSD |
| 59 | [102] | PSD of  δ, θ, α, β & γ | EEG | Mental Fatigue (drowsy prediction) | Driving simulator | Filters | WT for extracting Wavelet decomposition features.  Feature selection by LDA.  ANN as classifier |
| 60 | [62] | PSD of five bands, mean, median, variance, standard deviation, and mode from each of the frequency band | EEG | Mental Fatigue  (awake, drowsy and asleep) | Simulated driving | subtracting the noisy signal from the original EEG recording. | DWT & Debauchies’ wavelet |
| 61 | [141] | PSD of  δ, θ, α, β & γ;  TLI  and engagement Index  $\frac{\beta}{\alpha+\theta}$ | EEG | Vigilance due to Mental workload | Visual vigilance task | Notch filter | FFT using Neuron-Spectrum EEG software, |
| 62 | [83] | PSD of θ, α, and β relative power of them. The ratio of (α + θ)/β and fuzzy fusion ( α, β) | EEG | Mental Fatigue  (Vigilance, drowsy and sleep) | Real flight task | Filters | STFT by the Welch periodograms method (Hamming windows) |
| 63 | [64] | PSD of θ & α | 60 channels EEG | Mental fatigue | Monitoring task  (Detect color changes) | Filter | FFT for PSD |
| 64 | [66] | Absolute and relative PSD of α | 16 channels EEG & EOG | Mental fatigue | Simulated driving | Filters, EEGlab, and visual inspection. | FFT for PSD |
| 65 | [162] | PSD of θ & α | 16 channels EEG, ECG & EOG | Mental workload | Air Traffic control | Filters and Gratton method for eyes-blink artifacts | FFT for feature extraction & SWLDA for feature selection |
| 66 | [163] | PSD of θ & α | EEG | Mental workload | Air Traffic control | Band-pass filtered | FFT for feature extraction and SWLDA for feature selection |
| 67 | [84] | PSD of  δ, θ, α, β; Ratio of  θ/α, (θ + α)/β, (θ+α)/(α+ β), (α/ β) | 62 channels of EEG and two channels of EOG | Mental Fatigue  (alertness) | Simulated driving | ICA and filters | FFT for feature extraction  Fisher score technique selects the most descriptive and SVM as a classifier  distinguish the drowsiness level. |
| 68 | [239] | Average power and peak amplitude in the θ & α | 64 channels EEG & EOG | Mental fatigue | Long cognitive task (solving math problems on a computer) | Neuroscan Scan and low pass filter | Welch’s periodogram method for feature extraction of PSD  A kernel partial least squares as a classifier |
| 69 | [47] | PSD of α | 32 channel EEG &EOG | Mental fatigue | Memory workload task | Filters and ocular artifacts using a blind source separation algorithm | Frobenius distance and averaging in the region of interest |
| 70 | [160] | PSD of θ & α | One channel EEG, two channels ECG, three channels EMG &EOG | Mental workload and stress | Cement, traffic control, and power plant task while monitoring vital parameters of their systems. | Active noise cancellation technology applied by NeXus-4 data collection method. | NM |
| 71 | [221] | PSD | 128 channels EEG | Emotion recognition,  Stress &  mental  workload | maritime  simulator and EEG-based human factors evaluation in Air  Traffic Control | Filters | FFT for PSD  “CogniMeter system.” |
| 72 | [77] | PSD of θ, α & β | Eight channels EEG | Mental Fatigue  (vigilance detection) | Simulated driving | DWT for denoising | FFT and then converted to  a logarithmic scale. A Rectangle Window is used to extract PSD |
| 73 | [236] | PSD of δ, θ, α, β & γ | Seven channels EEG | Mental workload | Simulated  piloted aircraft with a secondary task  audio communication | Butterworth filter and ICA | STFT with Hanning window then RF for classifying  “adaptive system.” |
| 74 | [103] | PSD of θ, α, β & γ | 32 channels EEG & EOG | Mental fatigue | Simulated driving | Second-order blind by combining  ICA with entropy rate bound minimization (IC-A ERBM) | Welch spectrum of the PSD was used for converting the time-domain EEG data into the frequency domain.  AR for features extraction, and Bayesian neural network for classification. |
| 75 | [128] | PSD of θ & α | 128 channels EEG | Mental workload, Mental fatigue, and mental effort | Air traffic control | ICA from EEGLAB | STFT |
| 76 | [59] | PSD features for α & β | 64 channel EEG | Mental Fatigue  (vigilance) | Simulated driving | bandpass filter then ICA | STFT for PSD LSSVM and Particle swarm optimization classification |
| 77 | [72] | Log PSD of θ & α | 30 channels EEG | Mental Fatigue  (drowsy and alertness states) | Simulated driving | Filters, PREP pipeline (for bad channels removing) & ASR to reduce the high amplitude |  |
| 78 | [138] | PSD of δ, θ, α, β & γ | 56 channels EEG and ECG | Cognitive task workload | Monitor and control four air quality subsystems (O2 concentration, air pressure, CO2 concentration, and temperature). | Adaptive Filters and adaptive  exponential smoothing for removing motion artifacts | ICA and PCA  model |
| 79 | [126] | PSD of δ, θ, α, β & γ | 64 electrodes EEG, EOG, ECG, and NIRS | Mental Fatigue  (Drowsy & alertness) | Simulated driving | Visual inspection, ICA, low and high band filters | The locality preserving projection technique was utilized to process the EEG features  LSSVM is used as a classifier. |
| 80 | [142] | PSD of θ, α, β, & engagement index ratio  $\frac{\beta}{\alpha+\theta}$ | Two electrodes EEG | Mental Fatigue  (attention and cognitive resource engagement) | Simulated driving | No filtration | FFT for PSD |
| 81 | [179] | PSD of θ, α & β, and  engagement ratio  $\frac{average (\beta)}{average (\alpha)+average(\theta)}$ | 64 electrodes EEG | Task Engagement | Aviation | Filtered, underwent automatic channel rejection, and was cleaned using ASR | NM |
| 82 | [229] | The absolute PSD of α, β & γ; the relative power for θ; the power ratio of θ/ α | 24 channels EEG and ECG | Human error | Stroop test | NM | NM |
| 83 | [214] | The amplitude and latency of P300 | 15 channel EEG & EOG | Working memory load & attention | N-back task | Filters and  computerized  algorithm | Averaging ERP |
| 84 | [215] | The amplitude and latency of P300 | 27 channels  EEG | Working  memory and attention | N-back task | Filters | Averaging ERP |
| 85 | [210] | ERPs, evoked power & phase locking index | 19 channel EEG & 2 channel EOG | Working memory | Memory scanning tasks | NM | NM |
| 86 | [111] | The amplitude and latency of the N100, P200, P300, N200, MMN, and Nd. | EEG &EOG | Mental Fatigue  (attention) | Simulated aircraft landing task and  dichotic listening tasks | visual inspection | Averaging ERP |
| 87 | [213] | ERP components MMN,  P3a, and  Reorienting negativity  (RON) | 19 EEG | Working memory | Auditory duration  discrimination task | Filters | ERPs were computed within a time-window from -200-1000 ms |
| 88 | [134] | Amplitude and latency of P300 | EEG & EOG | Mental Fatigue | Visual display terminal task | Cross-correlation | Extracted by PCA |
| 89 | [131] | The amplitude and latency of ERN/Ne, N2, P3, amplitude & contingent negative variation  CNV | 22 channels EEG | Mental fatigue and motivation from task engagement | Monitoring task | Manual rejection for EEG, and Gratton Coles methods for EOG | Grand averages  Brain Vision Analyzer software |
| 90 | [185] | The amplitude of P2, P3, N1, N2, SW1, and SW2 | 19 channel EEG | Mental workload | Video Gaming | Manual rejection | ERP averages |
| 91 | [183] | The amplitude of P2, N2, P3b, CNV | 32 EEG EMG& EOG | Mental workload | A simulated driving task mainly Lane  Change Task with  a secondary auditory task | ICA | Averaging ERP |
| 92 | [186] | ERP Latencies of N1, P2, N2, P3, and MMN | Three EEG | Mental workload | Arithmetic task and reading comprehension | Filters | Averaging ERP |
| 93 | [187] | The amplitude N1, P2, P3, and LPP | 30 channels EEG | Mental workload | Gaming | An ocular artifact algorithm was applied to rely on regression analysis | Averaging ERP |
| 94 | [212] | The amplitude of P300 and P1 | 48 EEG & EOG | Working memory load | Sternberg memory task in addition to the flanker task | Offline analysis using EEGLAB | Averaging ERP |
| 95 | [130] | The amplitude and latency of P1, P2, P3b, N1& N2b | 62 channels  EEG | Mental fatigue | Practice blocks task visual selection task “Eriksen flanker task.” | Filters | Averaging ERP |
| 96 | [178] | The amplitude of P300 | Three EEG& EOG | Visual and mental workload | landing task and a classical oddball task. | Removed online | Averaging ERP |
| 97 | [180] | The amplitude of P300 | Three electrodes EEG | Cognitive Workload on attention | Air Traffic Control Aviation task and with an alarm detection task | high-pass filter and low-pass filter | EEGLAB for data analysis  Averaging ERP |
| 98 | [105] | The amplitude of P300 | 16 channel EEG | Mental Fatigue | Simulated driving | ICA | FFT for PSD  ANN as classifier  DBN is utilized to establish the evaluation model with four types of fatigue-based indicators. |
| 99 | [133] | The amplitude of P300 | 32 + 2 scalp sites EEG | Mental fatigue and task disengagement | N-back Task | Linear interpolation algorithm, Gratton and Coles method and averaging | Brain Vision Analyzer |
| 100 | [180] | The amplitude of P300 | 19 electrode EEG | Mental Workload | aviation task including an alarm detection task. | EEGLAB, Filters and visual inspection | Averaging ERP |
| 101 | [177] | The amplitude of P300 | EEG | Workload | visual search task | Filters and PCA | Averaging ERP |
| 102 | [129] | The amplitude of P3 and N2 | 60 active Ag/AgCl electrodes EEG | Time on task affects Mental fatigue | Simon task | Filters | Averaging ERP |
| 103 | [106] | ERP components and | 30 channels EEG | Mental fatigue | Simulated  driving | Bandpass filter | Time-frequency analysis using ERSP & inter-trial coherence (event-related phase-locking). Then CNN for prediction |
| 104 | [50] | The amplitude and latency of ERP components, namely N1& P3. | 30 electrodes EEG | Mental fatigue (attention) | Automated driving conditions vs. manual driving | Runica ICA algorithm and filters | Averaging ERP |
| 105 | [135] | EEG power in three bands: θ, α & β;  engagement index $\frac{\beta}{\alpha+\theta}$,  latency and amplitude of P300 ERP component | EEG | Mental fatigue (attention) | Tracking and display task. Auditory oddball task as a secondary task | Neuroscan software. | FFT for PSD |
| 106 | [70] | PSD of θ, α & β, and ERP components P1, N1, N2b | 30 channels EEG and EOG | Mental fatigue (attention) | Long visual attention task | Filter and Gratton and Coles method for EOG | FFT for PSD using a 100% Hanning window.  For ERP averaged time-locked |
| 107 | [68] | The amplitudes of ERP components N1, P2, and P300, power and peak amplitude of θ & α | 30 channels EEG, EOG & EMG | Cognitive fatigue | The mental arithmetic task from problem-solving | Manually rejected and filters | Welch’s periodogram method for PSD |
| 108 | [167] | The amplitude of P3, ERD/ERS α & θ | Seven electrodes EEG and EOG | Working load and memory | Attention and memory task | Filters | Brain Vision Analyzer software |
| 109 | [203] | PSD of β frequency and P700 ERP component | 32 channel EEG | Visual Fatigue | Watching an animation and then  random-dot stereogram | Filters and baseline correction | FFT for PSD |
| 110 | [169] | The absolute PSD values for  θ & α; the ratio of  $\frac{\theta}{\alpha}$, (θ * α), (θ + α), $\frac{l0g(\theta)}{\log(\alpha)}$; and the amplitude of P300 | Two-channel EEG | Cognitive Workload | Multitask (auditory, arithmetic, memory, and visual) | Ocular artifact reduction | FFT using Hanning window |
| 111 | [91] | The amplitude, peak frequency, and duration of θ spindle and amplitude of P3 | 128 EEG & ECG | Mental fatigue (attention) Vigilance | Simulated driving along with the auditory task | Filters and ICA | An automated algorithm to extract sharp spectral peaks within the alpha band.  For ERP averaged time-locked |
| 112 | [188] | The amplitude of P300, and PSD of θ & α | EEG& EOG | Mental Workload | N-back task | No artifacts were removed | Fourier analysis FFT for PSD  Grand averaging for ERP  SVM as classifier |
| 113 | [78] | PSD of δ, θ, α & β; the relative power, the amplitude, and latency of P300. | 32 EEG, EOG & ECG | Mental fatigue | Simulated driving | visually removed and filters | AR model with a 100% Hanning window |
| 114 | [182] | The amplitude and latency of P300, the PSD of θ & α | 31 electrodes EEG | Mental workload and fatigue | Dual-task (spelling task and listening task) | Filters | FFT for PSD |
| 115 | [52] | The amplitude of δ, θ, α, β; Ratio of  θ/α and (θ + α)/β  amplitude and SNR of  SSVEP | EEG and  Psychological measurement  Chalder Fatigue Scale | Visual fatigue | Visual task | Filters | FFT for amplitude  ANOVA for discrimination between alert and fatigue |
| 116 | [55] | Power of δ, θ, α, β & γ and amplitude of the N1, P2, N2, and P3 | 32 channels EEG & 2 electrodes EOG | Mental workload | Sternberg memory task | Butterworth filter and spatial filtering | Welch’s PSD |
| 117 | [216] | PSD of α, β, frontal θ, and the P300 amplitude  ERS/ERD % for θ, α & β | 27 electrode EEG & EOG | Working memory load | N-back, span and ospan tasks | ICA using EEGLAb, filter and visual inspection | FFT for band power  The ERD/ERS% were calculated for each data point with respect to a baseline. |
| 118 | [114] | Relative α band energy, Shannon’s, Rényi entropy second order and third order, and the ratio ($\frac{\alpha+\beta}{\delta})$ | 32 EEG, EOG, EMG&ECG | Mental Fatigue | Simulated driving and actual driving | Filtering and DWT | DWT |
| 119 | [190] | FD for emotions & PSD of δ, θ, α, β & γ for workload | 32 channels EEG | Stress,  Emotion &  Mental workload | Stroop Color-Word Test | Filters | FFT for PSD  Higuchi algorithm is used to calculate FD. Then extracted features are fed into the SVM to classify different starts. |
| 120 | [226] | FD, PSD of θ, α & β, statistical and  Higher-order crossings | 14 channel Emotiv EEG | Emotion | Emotion monitoring task | Filters | FD: Higuchi algorithm  PSD  Higher-order crossings: the second differences, mean of the absolute values  of the second differences of the normalized EEG |
| 121 | [81] | Time-domain: maximum, minimum, average, energy, and variance; complexity measures: sample entropy; Frequency domain: absolute and relative PSD of δ, θ, α, β & γ | EEG and ECG | Mental Fatigue (drowsiness & alertness) | Simulated driving | Visual inspection, Filtering using BESA software for eye blink artifact and EEGLAB software for movement artifacts | The classification was performed using the Weka data mining software implementing SVM |
| 122 | [42] | PSD of δ, θ, α & β  and ratios between them,  Non-linear features FD, SPEn, PmEn , & Generalized Hurst exponent | 16 channel EEG, ECG,  and photoplethysmography | Mental Fatigue (drowsy) | Simulated driving | Bandpass filter and notch filter | PSD: by using a Hanning window.  FD computed using the algorithm of Higuchi  Spectral entropy: derived in the normalized form  Permutation entropy: symbolized the elements of a time series.  Permutation entropy: derived from the relation between the rescaled range over the standard deviation  Classifiers: decision tree, RF, SVM linear function, SVM radial basis function, diagonal LDA & regularized LDA. |
| 123 | [108] | PSD of δ, θ, α, β & γ; mean, variance, zero-crossing rate; Shannon entropy, log-energy entropy, kurtosis.  The difference between θ, α, β & γ | 15 channel EEG | Mental fatigue | Air management system | ICA and bandpass filters | Dynamical deep extreme learning machine as a classifier  novel mental fatigue classifier |
| 124 | [156] | WT for θ, α & β | EEG | Mental workload | Monitoring task  (matching task) | Visual inspection | STFT |
| 125 | [99] | Zero crossing and integrated EEG for six different frequency scales | Six channels EEG | Mental fatigue (alertness and drowsiness) | Simulated driving | NM | Daubechies 2 stationary WT |
| 126 | [86] | WPE for θ, α & β; ratio of $\frac{\theta}{\alpha}$ , $\frac{\beta}{\alpha} , \frac{\alpha+\theta}{\beta}$, and Wavelet package entropy | 32 channels EEG, ECG & EOG | Mental fatigue | Mental arithmetic task | bandpass filter and adaptive filtering methods | WPE analysis  SVM as a classifier |
| 127 | [92] | WPE of the delta, theta, alpha, and beta along with the WPE ratio of  $\frac{\theta}{\alpha}$ , $\frac{\beta}{\alpha} and \frac{\alpha+\theta}{\beta}$ | 30 channel EEG, vertical EOG, and one channel ECG | Mental Fatigue | Simulated driving | Filters inspected visually, and then the possible residual artifacts were eliminated. | WPE analysis and FFT for extracting EEG signals and comparing them.  SVM- KPCA for feature classification |
| 128 | [117] | Entropy for δ, θ, α & β | EEG | Mental fatigue (transition from wakefulness to sleep) | Simulated driving | Filters | DWT, and then the entropy of each sub-band was calculated using Shannon entropy algorithm. Entropy was used to reduce the dimensionality of a feature vector.  The adaptive neuro-fuzzy inference system is developed as a classifier. |
| 129 | [26] | WE, PP-ApEn, and PP SampEn | EEG, EOG & EMG | Mental Fatigue | Simulated driving | Filtering & Nyquist’ rule | Entropy extracted using the WT  ANN as classifier |
| 130 | [113] | Approximate entropy and sample entropy | 14 EEG and EOG | Mental fatigue | Simulated driving | Adaptive filters for eye movement artifacts and visually for removing other artifacts | Entropies based feature extraction  SVM was applied to classify these two fatigue states. |
| 131 | [110] | SE, FE, AE & PE | 32 channel EEG | Mental fatigue (attention) | Simulated driving | Notch filter and bandpass filter Preprocessing using the software of Neuroscan. | Entropies based feature extraction  PE is calculated by Shannon function to the normalized PSD |
| 132 | [115] | Multiple Entropy Fusion including  spectral entropy, approximate entropy, sample entropy, and fuzzy entropy, | 32-channel electrode EEG | Mental Fatigue | Simulated driving | Bandpass filter | AR modeling, as feature extraction was employed in comparison with the multiple entropy fusion methods using training, and four classifiers were used: SVM, BPNN, RF, and KNN. |
| 133 | [88] | Alpha spindle and alpha power | 43 out of 62 EEG channels | Mental fatigue (awake vs. fatigued) | Simulated driving | ICA and AR | STFT for the spectral decomposition of |
| 134 | [90] | alpha spindle rate, alpha spindle duration, alpha & γ | 32 electrodes  EEG | Mental fatigue (attention) | In a simulated driving task (primary task) with two different secondary tasks  visuomotor task and  Auditory task | ICA using EEGLAB and auto-regression algorithm | FFT and Welch periodogram for PSD |
| 135 | [89] | Alpha spindle feature including alpha spindle rate and alpha spindle duration | 33 channels EEG | Mental fatigue (attention) | Simulated driving | ICA and bandpass filter | Features are extracted using  the full width at half maximum of the amplitude spectral density of the alpha frequency range algorithm.  Discounted autoregressive algorithm for discriminating |
| 136 | [225] | FD | 32 channel EEG | Emotions | Audio and visual stimuli | bandpass filter and DEAP benchmark database | DEAP for Feature extraction and SVM as a classifier. |
| 137 | [192] | FD and statistical features | 14 channels EEG | Mental workload, stress & Emotion | ship’s bridge virtual simulator | NM | The features are extracted using a sliding window of 4 seconds with a 75% overlap.  SVM as classifier |
| 138 | [120] | FD | EEG | Mental fatigue (arousal states) | Simulated driving | NM | FD using Grassberger Procaccia method. |
| 139 | [116] | FD, Detrended Fluctuation Analysis, Shannon entropy, Approximate Entropy, Sample Entropy, and Multiscale Entropy. | EEG | Mental fatigue (vigilance, drowsy and sleep) | Sleep task | Filters | Unsupervised classifier |
| 140 | [224] | FD and DWT for θ, α, β & γ | EEG &EOG | Emotion recognition | Displaying the Images task | bandpass filter. | DWT using Daubechies wavelet function  FD: by Higuchi’s algorithm correlation dimension: by  Grassberger and Procaccia algorithm  Feature selection using  Genetic Algorithm.  Classifier: Elman network which is a two-layer back-propagation neural network |
| 141 | [107] | Temporal and spatial covariance matrices calculated from EEG epochs | 64 channels EEG | Mental fatigue | A driving task in a VR environment. | Filters | Riemannian measure for classification. Convolutional neural network as a prediction method |
| 142 | [223] | RMS voltage for θ, α & β | EEG, EOG & EMG | Stress | Stroop color-word interference | Filters | k-NN  & logistic regression as a classifier |
| 143 | [95] | wavelet coefficients for δ, θ, α, β & γ | EEG, EMG, and EOG | Mental fatigue (vigilance) | Simulated driving | band-pass filter and ICA | CWT to extract the rhythm features of EEG data, while other features using STFT and the sparse representation method to the WT coefficient |
